# Bottom-Up Proteomic Analysis of Polypeptide Venom Components of the Giant Ant *Dinoponera Quadriceps*

**DOI:** 10.3390/toxins11080448

**Published:** 2019-07-29

**Authors:** Douglas Oscar Ceolin Mariano, Úrsula Castro de Oliveira, André Junqueira Zaharenko, Daniel Carvalho Pimenta, Gandhi Rádis-Baptista, Álvaro Rossan de Brandão Prieto-da-Silva

**Affiliations:** 1 Laboratory of Biochemistry and Biophysics, Instituto Butantan, São Paulo SP 05503-900, Brazil; 2 Laboratory of Applied Toxinology, CeTICS, Instituto Butantan, São Paulo SP 05503-900, Brazil; 3 Laboratory of Genetics, Instituto Butantan, São Paulo SP 05503-900, Brazil; 4 Laboratorio of Biochemistry and Biotechnology, Institute for Marine Sciences, Federal University of Ceara, Fortaleza CE 60165-081, Brazil

**Keywords:** *Dinoponera quadriceps*, Formicidae, Hymenoptera venom, proteomics, venom allergens, ICK-like toxins

## Abstract

Ant species have specialized venom systems developed to sting and inoculate a biological cocktail of organic compounds, including peptide and polypeptide toxins, for the purpose of predation and defense. The genus *Dinoponera* comprises predatory giant ants that inoculate venom capable of causing long-lasting local pain, involuntary shaking, lymphadenopathy, and cardiac arrhythmias, among other symptoms. To deepen our knowledge about venom composition with regard to protein toxins and their roles in the chemical–ecological relationship and human health, we performed a bottom-up proteomics analysis of the crude venom of the giant ant *D. quadriceps*, popularly known as the “false” tocandiras. For this purpose, we used two different analytical approaches: (i) gel-based proteomics approach, wherein the crude venom was resolved by denaturing sodium dodecyl sulfate-polyacrylamide gel electrophoresis (SDS-PAGE) and all protein bands were excised for analysis; (ii) solution-based proteomics approach, wherein the crude venom protein components were directly fragmented into tryptic peptides in solution for analysis. The proteomic data that resulted from these two methodologies were compared against a previously annotated transcriptomic database of *D. quadriceps*, and subsequently, a homology search was performed for all identified transcript products. The gel-based proteomics approach unequivocally identified nine toxins of high molecular mass in the venom, as for example, enzymes [hyaluronidase, phospholipase A1, dipeptidyl peptidase and glucose dehydrogenase/flavin adenine dinucleotide (FAD) quinone] and diverse venom allergens (homologous of the red fire ant *Selenopsis invicta*) and venom-related proteins (major royal jelly-like). Moreover, the solution-based proteomics revealed and confirmed the presence of several hydrolases, oxidoreductases, proteases, Kunitz-like polypeptides, and the less abundant inhibitor cysteine knot (ICK)-like (knottin) neurotoxins and insect defensin. Our results showed that the major components of the *D. quadriceps* venom are toxins that are highly likely to damage cell membranes and tissue, to cause neurotoxicity, and to induce allergic reactions, thus, expanding the knowledge about *D. quadriceps* venom composition and its potential biological effects on prey and victims.

## 1. Introduction

Ants (Vespoidea, Formicidae) belong to the class Insecta and the order Hymenoptera, which include the families Formicidae (ants), Apidae (bees), and Vespidae (wasps) among others [1]. The family Formicidae comprises more than 13,000 species of ants, most of which have an intricate social organization, form colonies with thousands of individuals, and are often distributed in every type of ecosystems on Earth [2]. The majority of ant species have a specialized venom apparatus to sting prey and victims, inoculating a cocktail of biological and pharmacological active substances for predation and defense [3]. The structure of the venom systems of ants share similar ontogenetic and evolutive relationship with the venom systems of wasps, which are characterized by free secretory tubules, a convoluted gland, and a venom gland reservoir [4]. The venom composition of several ant species has been investigated more recently at molecular and pharmacological levels, revealing unique repertories of toxins and venom-related polypeptides that differ considerably from the toxic components of other venomous animals. To mention a few examples, the venom polypeptide contents of the giant red bull ant *Myrmecia gulosa* [5], the bullet ant *Paraponera clavata* [6] and the fire ant *Solenopsis invicta* [7,8]. Ant venoms are rich in organic compounds (e.g., alkaloids and amines), short linear peptides, allergens, and hydrolases, with a very few representatives of cysteine-stabilized peptides [9,10]. In this respect, the peptide composition profile of ant venom is intriguing and a rare exception within the field of venomics, given that most animal venoms contain distinct families of toxins with variable number (from 1 to 5) of internal disulfide bonds that contribute to polypeptide stability and potency [11,12].

The study of venom composition of certain group of animals is advantageous, since it advances (*i*) the overall understanding of the outcome of the pathophysiological process of envenomation, (*ii*) the isolation of a given group of toxins for determining structure-activity relationship and (*iii*) the development of lead compounds for clinical application from particular structures with intrinsic biological and pharmacological activities, among other factors of interest to biomedicine and pharmaceutical biotechnology. For instance, from the venom of the Israeli scorpion *Leiurus quinquestriatus*, a chloride-channel peptide inhibitor with insecticidal and cell-penetrating properties, has been investigated to treat glioma and other types of cancer [13]; from the Chinese red-headed centipede *Scolopendra subspinipes mutilans*, a novel selective peptide inhibitor of voltage-gated sodium channel (subtype 1.7), which could be converted into a lead compound for the development of analgesics, was characterized [14]. Considering that arthropods, particularly ants, are the predominant venomous species on Earth and the large majority of venomous species and their venoms have been comparatively scarcely investigated, a considerable diversity of venom-derived polypeptide structures and their intrinsic biological activities remain to be potentially discovered and characterized.

Among all known subfamilies of ants, the *Ponerinae* subfamily comprises a group of primitive, venomous and one of the largest species (~ 3 cm) of ants in the world found in tropical regions of the Earth and distributed mainly in the Amazon basin. The genus *Dinoponera* groups together eight described carnivorous species, collectively referred as “false tocandiras”, in contrast to the bullet ant *P. clavata* (“tocandiras”). Like most ant species, the predatory *Dinoponera* giant ants utilize their venom, delivered by a sting, to hunt prey (insects, small birds, and mammals) and defend themselves against aggressors (microbials and predators) [2,15]. Accidental and fortuitous encounters with humans occur, but in such circumstance the extremely violent sting, aside from causing long-lasting local pain, provokes systemic symptoms, like fever, involuntary shaking, cold sweating, nausea, vomiting, lymphadenopathy, and cardiac arrhythmias [16]. The giant ant *D. quadriceps* (Santschi, 1921; Kempf, 1971) is distributed and found in the remains of the Atlantic forest, upland humid forests, and the Cerrado and Caatinga biomes in the northeastern region of Brazil, where xerophytic vegetation and low pluviometry regime prevails [15]. Previous studies have described numerous biological and pharmacological activities of the *D. quadriceps* crude venom, such as anti-coagulant, anti-inflammatory and anti-platelet activities and induction of allergic reactions and possible anaphylaxis [17]. The antinociception, anticonvulsant, neurotoxic and/or neuroprotection [18,19], as well as antimicrobial and anti-parasitic affects, have also been reported [20,21]. It is interesting to note that traditional knowledge advocates the use of these ants (and venom) in the treatment of asthma, rheumatism, ear pain, and back pain [22]. In fact, poneratoxin-like peptides obtained from the parental species *P. clavata* were shown to slowly induce the activation of ion currents in response to small depolarization steps and sustain currents due to inactivation blockage of the Nav1.7 channel [6], consequently serving to modulate neurotransmission.

In one of our recent works, a complete analysis of the venom gland transcriptome from *D. quadriceps* was reported [23]. The venom-derived polypeptide and toxin precursors were categorized in two main core components: a major core of predicted toxins that comprises venom allergens (homologous to Sol i 1/PLA1B, Sol i 2/4, and Sol i 3/Ag 5), lethal-like proteins, and esterases (phospholipases A and B, acid phosphatases, and carboxylesterase) and dinoponeratoxins; a minor group of components, which include conserved inhibitory cysteine knot (ICK)-like toxins and comprise, to date, one of the rare examples of this class of cysteine-stabilized toxin in the venom from ants, together with an ICK-like peptide from the venom of the Asian ant *Strumigenys kumadori* (Myrmicinae) [9]. Some of these toxin precursors, like dinoponeratoxins, are homologous to the peptide toxins present in the venom of other *D. quadriceps* populations [24] and in other giant ant species, such as *D. australis* [25]. Allergens are also conserved venom components retrieved from the *D. quadriceps* transcriptome that had been observed only in ant species outside the *Dinoponera* group, mainly in the fire ant *S. invicta* (Myrmicinae), from which the allergen nomenclature is derived (e.g., Sol 1, 2, 3, 4) [26].

The bottom-up proteomics approach involves the chemical or enzymatic cleavage of intact proteins into small peptides that are subsequently backtracked to their original structure and/or set of proteins (proteome). In this proteomic approach, proteins are resolved by one- or two-dimensional gel electrophoresis, after which the bands or spots are excised, in-gel digestion is achieved, and the resulting proteolytic (tryptic) peptides are analyzed by liquid chromatography–mass spectrometry (LC–MS). Additionally, the in-solution digestion of the total protein content in a homogeneous sample, like crude venom, is performed, and the resulting tryptic peptides are analyzed by LC–MS; this is a direct, associated approach [27].

Based on these facts and considering that the transcriptome of *D. quadriceps* venom gland predicted a predominant venom component not completely and functionally characterized yet, the aim of this work was to examine the polypeptide toxins of *D. quadriceps* expressed in the venom, by means of a bottom-up proteomic analysis, including in gel- and in-solution venom proteomics and comparison of toxin identifiers with products encoded by the transcripts expressed in *D. quadriceps* venom gland.

## 2. Results

### 2.1. Bottom-Up Proteomics of Dinoponera quadriceps Venom

**Figure 1:**
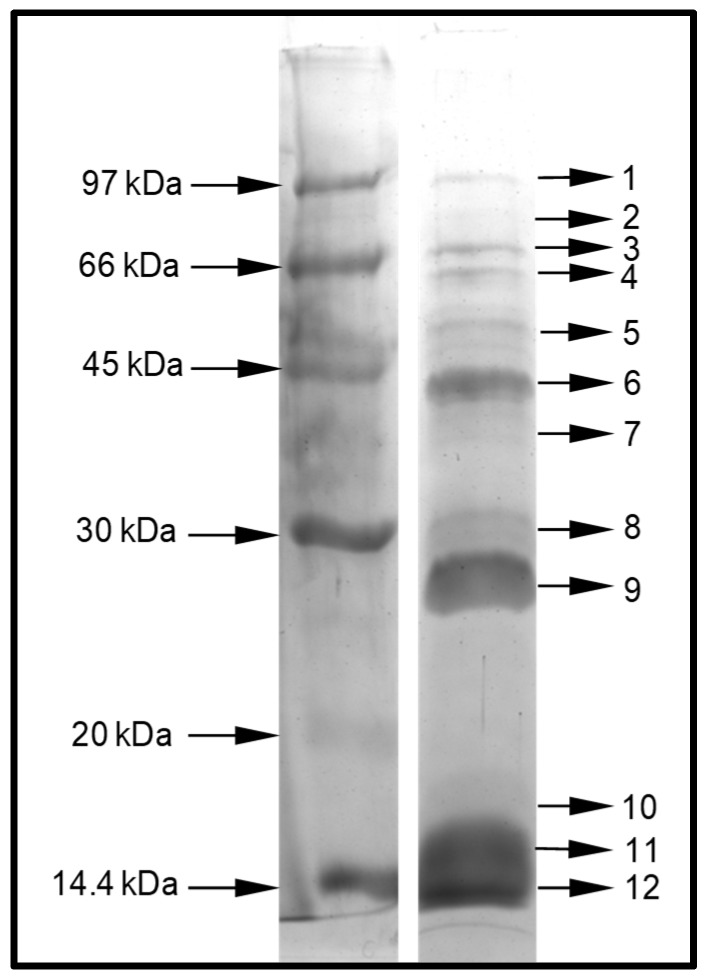
Resolution of *D. quadriceps* venom by denaturing sodium dodecyl sulfate -polyacrylamide gel electrophoresis (SDS-PAGE). Dried venom (30 µg) was solubilized in SDS-PAGE sample buffer and separated in 12.5% T/2.6% C SDS-PAGE, under reducing conditions. The Coomassie stained SDS-PAGE revealed the presence of 12 major, predominant bands in the crude venom. These bands were excised and submitted to an in-gel digestion protocol. The tryptic peptides (obtained for each band) were analyzed by liquid chromatography–electrospray ionization–mass spectrometry (LC-ESI-MS/MS) and the output data were analyzed against a transcriptomic database of *D. quadriceps* venom gland. The digitalized image was converted to black and white. Relative molecular mass is indicated in the left side. Bands of resolved venom toxins are numbered in the right side.

We studied the protein content of *D. quadriceps* venom using two proteomic strategies: (1) in-gel digestion and (2) in-solution digestion, followed by analysis with two different mass spectrometers. Initiating with the first strategy, as shown in Figure 1, 12 major clear protein bands were observed in the denaturing sodium dodecyl sulfate-polyacrylamide gel electrophoresis (SDS-PAGE). These bands were excised from the polyacrylamide gel, submitted to an in-gel digestion procedure, and analyzed by liquid chromatography coupled to a mass/mass spectrometer (LC–MS/MS). The output data were compared with the transcriptomic database of the *D. quadriceps* venom gland. The list of venom proteins identified as toxins is presented in Table S1.

Our gel-based proteomics analysis resulted in the unequivocal identification of nine major venom components that match perfectly with their respective transcripts (contigs) expressed in the *D. quadriceps* venom gland (Table S1). After performing a homology search of databases, using the basic local alignment search tool (BLASTx), these main venom components were matched with the following polypeptides resolved by SDS-PAGE: gel band 1: venom dipeptidyl peptidase 4 (XP_014479089.1); gel bands 3 and 4: glucose dehydrogenase flavin adenine dinucleotide (FAD) (XP_014475855.1); gel band 5: protein yellow-like (XP_014473983.1); gel bands 6, 7, 8, 10, and 11: phospholipase A1 2-like (XP_014477282.1); gel band 7: hyaluronidase-like (XP_014477391.1); gel band 9: venom allergen 3-like (XP_014469499.1). In addition, we found three uncharacterized components: (i) gel bands 10, 11, and 12 corresponding to an uncharacterized protein LOC106750875 (XP_014486970.1); (ii) gel band 11 representing an uncharacterized protein LOC106751228 (XP_014487552.1); (iii) gel bands 11 and 12 representing an uncharacterized protein LOC105189919 (XP_011150625.1). Peptide fragments from these three uncharacterized proteins revealed a toxin with a Sol_i_2 (pheromone-binding protein/odorant-binding protein, OBP/PBP) domain (Table S1). Only in gel band 2, the giant ant venom-protein was not accurately identified. The complete list of venom polypeptides and their correspondent transcriptomic contigs that were identified through gel-based proteomics approach is shown, as aforementioned, in Table S1.

The second strategy employed to study the *D. quadriceps* venom was the solution-based proteomics approach, followed by analysis in two different mass spectrometers. This approach resulted in the identification of peptides corresponding to 61 venom-related transcript products in “analysis #1” (realized with the LTQ-Orbitrap Velos mass spectrometer) and 87 transcript products in “analysis #2” (realized with the Q Exactive Plus mass spectrometer) (Table S2). Both in-solution analyses led to the identification of 44 transcripts, of which seven were also identified in the in-gel analysis. These seven transcripts were venom dipeptidyl peptidase 4, glucose dehydrogenase FAD, protein yellow-like, phospholipase A1 2-like, hyaluronidase-like, venom allergen 3-like, and uncharacterized protein LOC106750875. These results reinforce the gel-based proteomics strategy, and also indicate the presence and expression of several protein components in the venom in quantities that is out of the dynamic range of Coomassie brilliant blue (CBB)-staining of venom proteins separated by denaturing SDS-PAGE. For example, the results from these in-solution proteomic analyses showed the presence of less abundant polypeptides visible in the CBB-stained SDS-PAGE, such as hydrolases (acidic phospholipase A2 PA4, arylsulfatase J, carboxypeptidase Q-like, cathepsin L1, lysozyme c-1-like, putative cysteine proteinase CG12163, venom acid phosphatase Acph-1-like, venom serine protease-like), oxydoreductases (superoxide dismutase [Cu-Zn]), protease inhibitor (serpin B3-like, Kunitz-type serine protease inhibitor ki-VN-like) or proteins related to lipid metabolism (apolipophorin-III, prosaposin, protein NPC2 homolog). A list of venom polypeptides and their corresponding transcriptomic contigs that were identified through solution-based proteomics are present in Table S2.

### 2.2. Comparison of Identified Venom-Derived Polypeptides

All proteins identified in the *D. quadriceps* venom were classified according to their typical functional and/or structural domains (structural protein units) (Tables S1 and S2), based on our previous transcriptome analysis, gene and genome databanks, and the Pfam library database. In the following sections a description of each of the predominant polypeptide component of the *D*. *quadriceps* crude venom is presented.

#### 2.2.1. Venom Dipeptidyl Peptidase-4 (vDPP-4)

**Figure 2:**
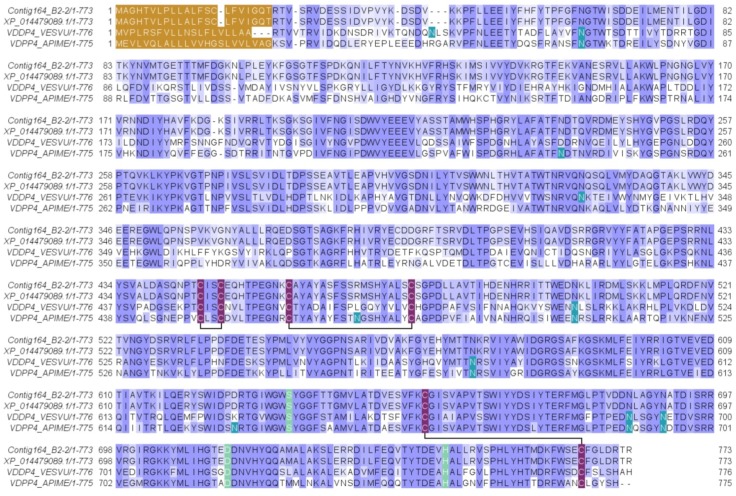
Alignment of the *D. quadriceps* dipeptidyl peptidase-4 (vDPP-4) encoded in the transcript Contig164_B2-2 and sequences of homologous proteins from common wasp and honey bee. The *D. quadriceps* vDPP-4 was aligned with its homologues from common wasp *V. vulgaris* (VDDP4_VESVU), honey bee *A. mellifera* (VDPP4_APIME) and a predicted sequence from the genome of *D. quadriceps* (XP_014479089.1). Asparagine residues (N), marked in emerald green, indicate glycosylation sites. The light green tagged amino acids indicate residues located at the enzyme active site. The cysteine residues that are involved in the formation of disulfide bridges are indicated in purple and the connectivity pattern is indicated by solid black lines. The peptide signals are labeled in brown and were predicted using SignalP 5. The conservation is indicated by BLOSUM62 matrix using the Jalview program.

**Figure 3:**
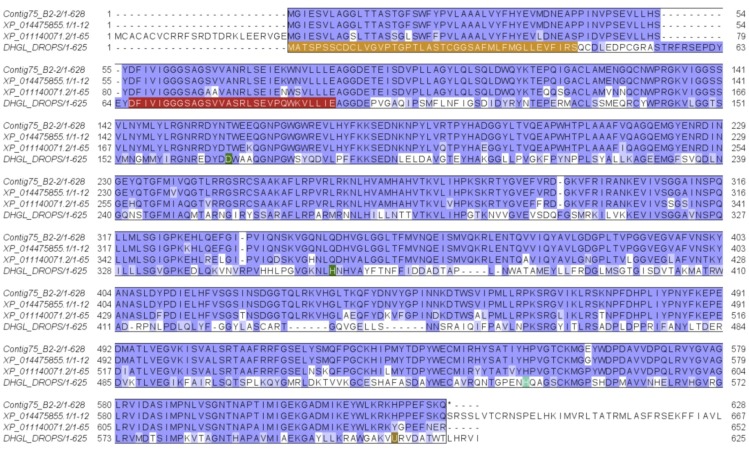
Alignment of *D quadriceps* venom glucose dehydrogenase [FAD, quinone] with homologous proteins from hymenopterans. This venom component identified by proteomic analysis of band 3 and 4, resolved in the SDS-PAGE, is encoded in the *D. quadriceps* venom gland transcript contig75_B2-2 was aligned to the N-terminal (the first 612 residues) of the predicted sequence generated by automatic annotation of *D. quadriceps* genome (XP_014475855), the Indian jumping ant *H. saltator* (XP_011140071) and the Glucose dehydrogenase [FAD, quinone] from fruit fly *D. pseudoobscura* (DHGL_DROPS). The *Drosophila* protein (DHGL_DROPS) comprises a peptide signal of 42 amino acids (experimental). The nucleotide binding site is indicated in red. The amino acids that are indicated in green are divergent or conflictive residues. Selenocysteine is indicated by “U”.

This protein was identified from gel band 1 (with peptide fragments covering 1% of the full sequence) and from the in-solution proteomic analysis protein 10 (with peptide fragments covering 44% and 54% of the full sequence). This venom-protein corresponds to the transcript contig164_B2-2 of the *D. quadriceps* venom gland transcriptome previously reported [23]. This transcript encodes a precursor of which the protein product, in the venom, is homologous to human dipeptidyl peptidase 4 (vDPP-4) or CD26. Such venom enzyme is also known as allergen C (Api m 5) in the honey bee *Apis mellifera* and Ves v 3 in common wasp *Vespula vulgaris* [28]. The organization of the cDNA precursor and the alignment of the *D. quadriceps* vDPP-4 (Venom allergen C) with its homologues from bee and wasp venoms are shown, respectively, in Figure S1 and Figure 2. Here, in *D. quadriceps*, the precursor consists of a signal peptide with 21 amino acid (aa) residues and a mature protein with 752 aa with a theoretical molecular mass of 85842.88 Da (Figure S1 and Figure 3). The vDDP-4 encoded by Contig164_B2-2 of the *D. quadriceps* transcriptome [23] has predictive glycosylation sites at the same positions of the best studied sequences of bee and wasp vDPPs, which cause an apparent distinct electrophoretic mobility on SDS-PAGE of approximately 100 kDa (Figure 1). A list of vDPPs in the Hymenoptera database can be found in the Peptidase S9B family and DPP-4 subfamily.

#### 2.2.2. Glucose Dehydrogenase [FAD, Quinone]

This protein was identified from the SDS-PAGE gel bands 3 and 4 (with peptide fragments covering 6% and 3% of the full sequence), from the in-solution proteomic analysis protein 13 (with peptide fragments covering 34% and 45% of the full sequence), and from the protein precursor encoded in the *D. quadriceps* transcript contig75_B2-2, a venom polypeptide of the FAD family of flavoprotein oxidoreductases. Its sequence is structurally similar to other hymenopteran proteins, such as the *glucose dehydrogenase* [FAD, quinone] of the jumping (or Jerdon’s) ant *Harpegnathos saltator* ant (XP_011140071.2) and fruit fly *Drosophila pseudoobscura* (DHGL_DROPS). These venom polypeptide sequences exhibit highly conserved sites for binding nucleotides and enzymatic catalysis (Figure 3). The protein identified in this band is also highly similar to the N-terminal (580 aa) of a duplicated genome-encoded sequence of *D. quadriceps* (XP_014475855) that predicts a polypeptide of 1271 aa. The organization of the *D. quadriceps* precursor of venom glucose dehydrogenase (GDH) [FAD, quinone] is shown in Figure S2. Owing to the presence of glycosylation sites, proteins bands (3 and 4) appear to migrate in SDS-PAGE with an apparent molecular mass of 55 to 65 kDa, instead of the theoretical molecular mass of 65379.84 Da. A list of similar polypeptides in the Hymenoptera database can be accessed in the entry family of glucose-methanol-choline (GMC) oxidoreductases.

#### 2.2.3. Yellow Royal Jelly Protein Domain

**Figure 4:**
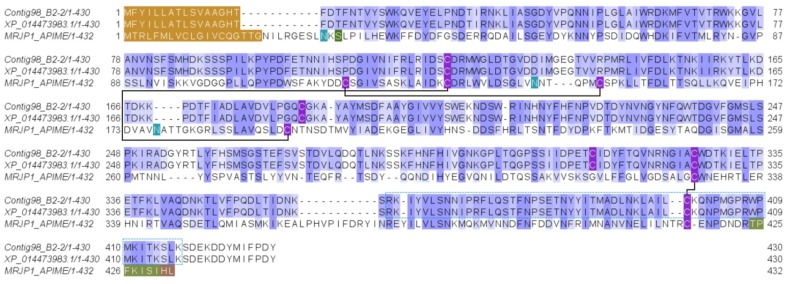
Alignment of *D. quadriceps* major royal jelly protein (MRJP)-like precursor to predicted and known similar sequences. MRJP-like protein identified initially in the *D. quadriceps* venom gland transcriptome, confirmed by the proteomic analysis of the crude venom (this study), was aligned to its counterparts in *D. melanogaster* [XP_014473983] and honey bee *A. mellifera* [MRJP1_APIME]. Pattern of conservation is indicated using the JalView program with the BLOSUM62 matrix. The signal peptides of MRJP-sequences are indicated in brown. The amino acid marked in green are divergent or conflictive residues. Asparagine residues are marked in light green and they indicate glycosylation sites. The MRJP-1 regions which generate the antimicrobial peptides Jelelin-1 to -4 are indicated in dark green. The cysteine residues are highlighted in purple and the pattern of MRJP-1 disulfide bonds is indicated by connecting lines.

This protein was identified from the SDS-PAGE gel band 5 (with peptide fragments covering 8% of the full sequence) and from the in-solution proteomic analysis protein 10 (with peptide fragments covering 56% and 70% of the full sequence). It was identified as the major royal jelly protein domain [MRJP domain], a 215-aa venom polypeptide corresponding to the transcript contig98_B2-2 (Figure 4 and Figure S3). This transcript (1614 bp) encodes a 430-aa long MRJP precursor, which has a signal peptide (16 aa) and a mature venom protein (414 aa) (Figure S3), with a theoretical molecular mass of 47616.06 Da and an apparent electrophoretic mobility of 45 kDa on SDS-PAGE (Figure 1, gel band 5). A list of proteins from this family in the Hymenoptera database can be found under the entry “major royal jelly protein family”. The transcribed yellow protein-like protein of *D. quadriceps* (Figure 4) shares 27% similarity with the *A. mellifera* MRJP-1 protein and 40% identity with the *D. melanogaster* yellow protein. The honey bee-like glycoprotein MRJP-1 is a 55 kDa monomer, but forms oligomers of 420 kDa [29]. The MRJP-1 from honey bee has three pairs of cysteine bonds, whereas the MRJP-like protein from *D. quadriceps* venom contains only five residues of cysteine, possibly forming only two disulfide bonds.

#### 2.2.4. Phospholipases A1 (PLA1)

**Figure 5:**
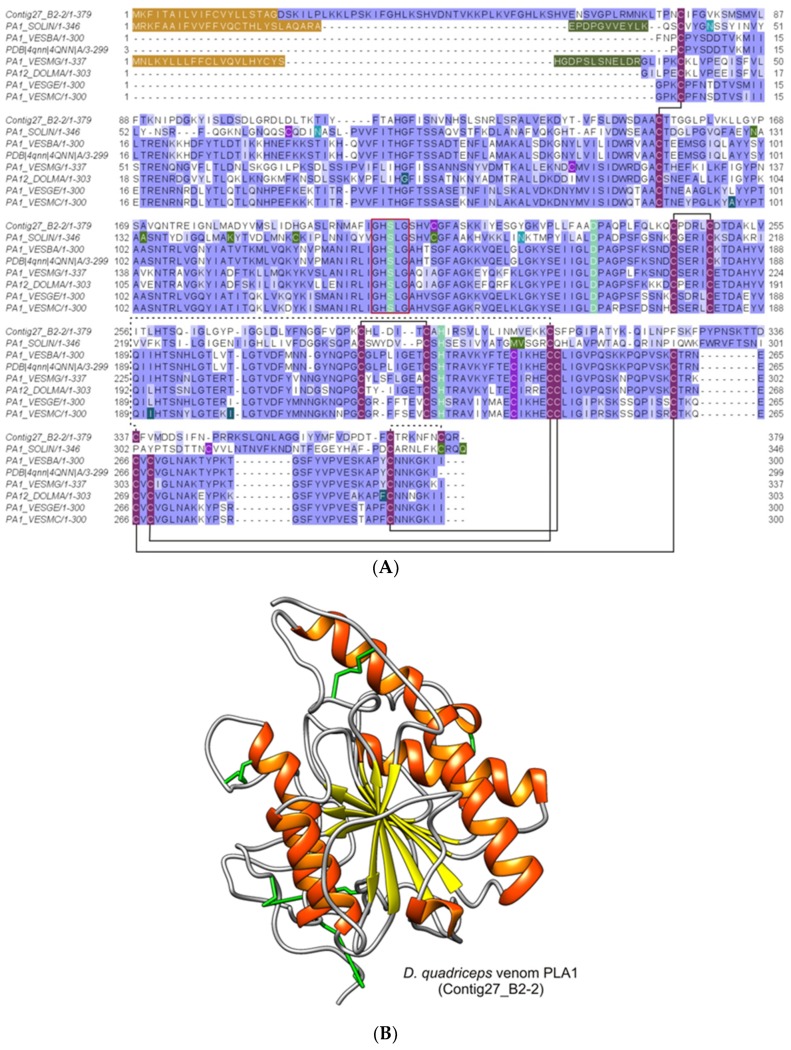
Alignment and structural model of *D. quadriceps* venom phospholipase A1. (**A**) *D. quadriceps* venom phospholipase A1 was aligned to some hymenopteran homologues: [PA1_SOLIN], PLA1 (Allergen Sol i 1) from *S. invicta*; [PA1_VESBA], Phospholipase A1 from black-bellied hornet *Vespa basalis* and [PDB | 4QNN | A], sequence that generated the crystal structure of the A chain by X-ray diffraction; [PA1_VESMG], hornet wasp *Vespa magnifica* phospholipase A1 magnifin; [PA12_DOLMA] phospholipase A1-2 (Allergen Dol m 1) from bald-faced hornet *Dolichovespula maculata*; [PA1_VESGE] phospholipase A1 (Allergen Ves g 1) form European wasp (German yellowjacket) *Vespula germanica*; [PA1_VESMC], phospholipase A1 (Allergen Ves m 1) from Eastern yellow jacket *Vespula maculifrons*. Glycosylated asparagine residues are indicated in emerald green. Dark green amino acids indicate natural variants. The amino acids from the enzymatic active site are indicated in light green and the consensus Gly-X-Ser-X-Gly motif, characteristic of the active serine hydrolases, is boxed in red. Moss green indicated divergent or conflictive residues. The signal peptide is indicated in brown and the pro-peptide in military green. Conserved cysteine residues that participate in the formation of disulfide bridges are indicated by purple boxes and connected by solid black lines, as determined for wasps’ PLA1s. Unpaired cysteines are indicated in light purple and the connecting lines indicate the possible connectivity based on homology model of the mature PLA1 sequence of *D. quadriceps*. The conservation was estimated with the BLOSUM62 matrix. (**B**) Structural model of *D. quadriceps* PLA1 predicted by homology modeling from venom gland transcript Contig27_B2-2 and the venom phospholipase A1 from hornet wasp *Vespa basalis* (PDB 4QNN), as template. This giant ant venom component was detected in-gel analysis (gel bands 6 and 7). Similarly, predicted from the transcripts consensus_20 Phospholipase A1-like 1_B04_E7_DVC2, that corresponds to gel bands 10 and 11. Also detected in-solution proteomic analysis: protein 7 (Contig27_B2-2); protein 17 (1_B04_E7_DVC2); Based on the structural analysis, this giant ant toxin is presumably a platelet activator, like in Vespidae venoms. Note: protein 12, from in-solution analysis, is a PLA2.

These proteins were identified from gel bands 6, 7, 8, 10, and 11 (with peptide fragments covering 19% of the full sequence) and from the in-solution proteomic analysis protein 7 (with peptide fragments covering 51% and 65% of the full sequence). They correspond to the phospholipase A1 homologues, the polypeptide products of the *D. quadriceps* venom gland transcript contig27_B2-2. These peptides and transcript products also correspond to a predicted partial sequence transcribed and translated from the *D. quadriceps* genome segment (XP_014477282). The polypeptide is similar to the venom allergen 1 of *S. invicta,* sharing 51% identity. The *D. quadriceps* PLA1 homologue, encoded by contig27_B2-2 (1656 bp), is a preprotein of 379 residues and a mature polypeptide with 356 aa residues (theoretical molecular mass 38715.85). The structural organization of transcript contig27_B2-2 and its polypeptide product can be seen in Figure S4. As observed in the SDS-PAGE gel (Figure 1) and peptide mass fingerprinting (PMF) from the in-gel digestion of corresponding bands (Table S1), the *D. quadriceps* PLA1 has several post-translational modifications (PTMs) that interfere with the electrophoretic migration on SDS-PAGE. The identified venom PLA1 of *D. quadriceps*, aligned with other hymenopteran sequences, is shown in Figure 5. A list of Hymenoptera phospholipases that belong to the alpha/beta (AB) hydrolase superfamily (lipase family) can be found in the Hymenoptera database.

#### 2.2.5. Hyaluronidase

**Figure 6:**
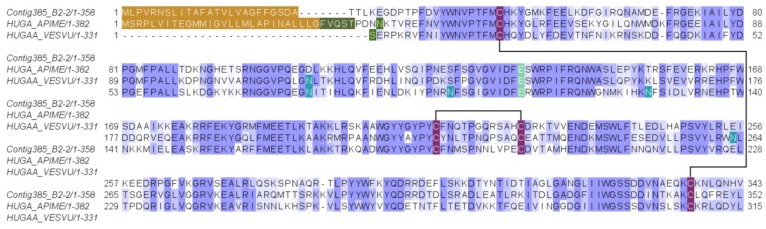
Alignment of the venom *D. quadriceps* hyaluronidase and homologous enzymes from hymenopterans. *D. quadriceps* venom hyaluronidase, encoded in the venom gland transcript contig385_B2-2 and identified by in-gel and in-solution proteomics analysis, is compared with similar proteins from honey bee *A. mellifera* [HUGA_APIME] and common wasp *V. vulgaris* [PHUGAA_VESVU]. Signal peptide is boxed in brown color and the prepropeptide in dark green. Glycosylation residues (N) are in blue. Conserved cysteine residues are indicated in purple color and the disulfide bonds by connecting solid black lines.

This protein was identified from gel band 7 (with peptide fragments covering 3% of the full sequence) and from the in-solution proteomic analysis protein 16 (with peptide fragments covering 16% of the full sequence). The peptides detected in the venom proteome corresponded to the translated protein product of the *D. quadriceps* venom gland transcript contig385_B2-2 (1882 bp, Figure S5), which codes for the venom hyaluronidase that belongs to the family of glycosyl hydrolases 56 in the Hymenoptera database. Such sequence is identical to the predicted product of the gene segment of *D. quadriceps* genome [XP_014477391]. The protein precursor of *D. quadriceps* hyaluronidase is 368 aa long, with a signal peptide of 25 residues and a mature protein of 343 aa (theoretical molecular mass 38844.96 Da), but the glycosylation-specific sites, which could cause the apparent distinct pattern of electrophoretic migration to diverge from the theoretical molecular mass, are identifiable (Figure 1, Table S1). The *D. quadriceps* venom hyaluronidases share high similarity (99%) with homologous sequences from the venoms of common wasp and honey bee (Figure 6).

#### 2.2.6. Major Venom Allergen 3, Cysteine-Rich Venom Protein Superfamily; Cysteine-Rich Secretary Protein (CRISP) Family

**Figure 7:**
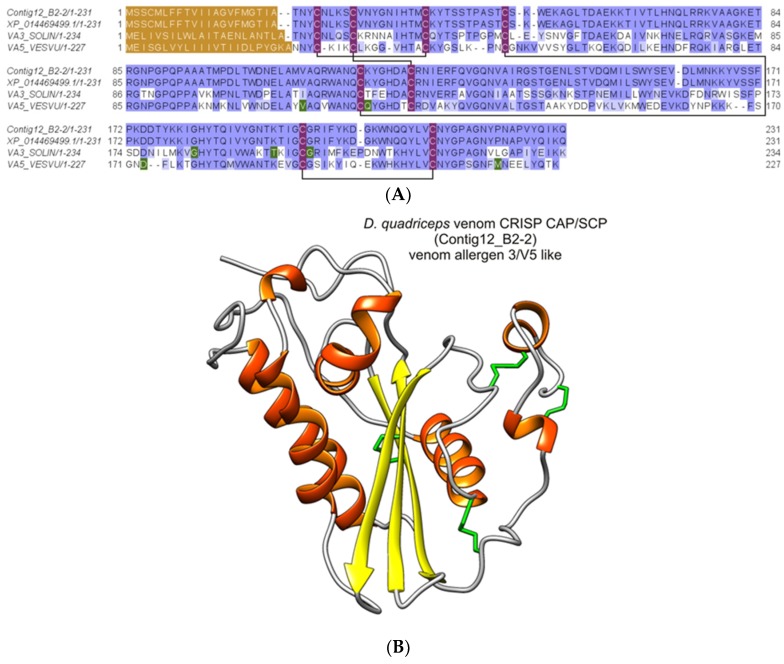
Alignment and structural model of cysteine-rich venom protein/major venom allergen 3 from *D. quadriceps* venom. (**A**) the *D quadriceps* venom CRISP-, CAP/SCP-like protein was aligned to its hymenopteran homologues. In this alignment, proteins were compared with the predicted protein from the gene segment of *D. quadriceps* genome [XP_014469499.1] (venom allergen 3-like), venom allergen 3 of *S. invicta* [VA3_SOLIN] and venom allergen 5 of *V. vulis* [VA5_VESVU]. (**B**) Structure of *D quadriceps* venom CRISP-, CAP/SCP-like protein that was modelled from the sequence predicted from *D. quadriceps* venom gland transcript Contig12_B2-2, using as template the crystal structure of the major allergen Sol i 3 from fire ant venom *Solenopsis invicta* (PDB 2VZN). The *D. quadriceps* CRISP-, CAP/SCP-like venom protein was identified by in-gel proteomics (gel band 9), and in-solution proteomics, proteins numbered 1, 8 and 67, which correspond to venom gland transcripts 1_A09_D9_DVA2-1; 1_A09_D9_DVA2; 1_C05_C6_3_DVB1; Consensus TX03; 1_E07_H11_DVC2-1; 1_E07_H11_DVC2; 1_B07_E6_DVC2-1; 1_A09_D9_DVA2-1; 1_A09_D9_DVA2; 1_E06_B4_DVC2-1; 1_E06_B4_DVC2; 1_B03_C4_2_DVC2; 1_C01_H9_3_DVC2. By comparative analysis with the major venom allergen 5 from vespoid wasps, the venom allergen 3 from fire ants and the scoloptoxins from the Thai centipede *Scolopendra dehaani*, which cause allergic reactions after stinging, this venom-protein in *D. quadriceps* is presumed to be a potent allergen.

This protein was identified from the SDS-PAGE gel band 9 (with peptide fragments covering 23% of the full sequence) and from the in-solution proteomic analysis protein 16 (with peptide fragments covering 52% and 47% of the full sequence). It corresponded with the major venom allergen 3, a cysteine-rich venom protein precursor encoded by the *D. quadriceps* venom gland Contig12_B2-2 (Table S1). This protein belongs to the sperm-coating glycoprotein (SCP)/cysteine-rich secretory proteins (CRISP), antigen 5, and pathogenesis-related 1 proteins (CAP) superfamily that also includes cysteine-rich venom proteins that together make up 9 subfamilies of proteins. The 210-aa long mature protein product of the *D. quadriceps* transcript (molecular mass 23660.77 Da) could be glycosylated (Figure S6), as shown by the change in its electrophoretic mobility. Similar sequences in hymenopteran venom share high level of similarity, for example, venom allergen 3 [P35778] from *S. invicta* (Antigen Sol i 3) with 57% identity and major antigen of wasps (Vespid venom allergen V5) (Figure 7).

#### 2.2.7. Major Ant Venom Allergen 2/4-Like (Odorant/Pheromone-Binding Protein-Like)

**Figure 8:**
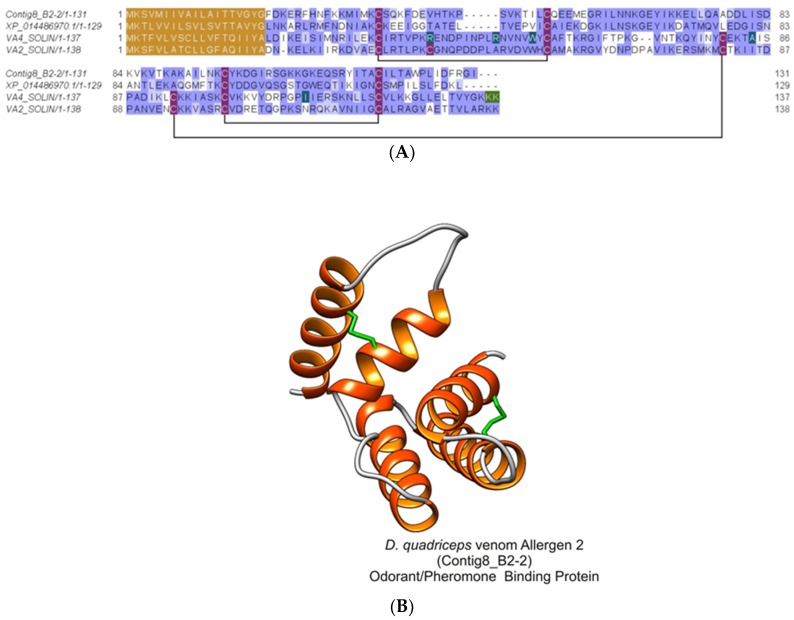
Structural analysis of *D. quadriceps* ant venom allergen 2/4, pheromone/odorant binding protein-like (OBP/PBP). (**A**) Alignment of *D. quadriceps* OBP/PBP-like and venom proteins Sol i 2 and Sol i 4 from fire ant *S. invicta*. The protein product of *D. quadriceps* transcript (Contig8_B2-2) expressed ion the venom gland, confirmed by proteomic analysis of the crude venom and the predicted amino acid sequence from the genomic annotation of *D. quadriceps*, XP_014486970.1, as well as the venom allergen 2 (Sol i 2) [VA2_SOLIN] and venom allergen 4 (Sol i 4) [VA4_SOLIN] from *S. invicta* were aligned. In brown, the leader sequences are shown as predicted by SignalP 5.0. In dark green, natural variants, conflictive or divergent residues are indicated and in light green. In purple, the conserved cysteine residues are highlighted. The solid black lines indicate the pattern of disulfide bonds, as determined for Sol i 2 [VA2_SOLIN]. Amino acid conservation are estimated with the BLOSUM62 matrix. (**B**) Structural model of *D. quadriceps* venom allergen 2/4, OBP/PBP-like venom protein. The predicted structure was homology modelled from *D. quadriceps* contig8_B2-2, based on the crystal structure of the venom allergen Sol i 2 from fire ant *S. invicta* (PDB 2YGU), as template. This *D. quadriceps* venom component was detected by in-gel analysis (gel bands 10, 11 and 12) and by in-solution proteomic analysis (protein 3 that corresponds to transcript Contig8_B2-2) and similar sequences, like protein 2 (1_B07_F8_2_DVB1); protein 4 (1_C11_G4_3_DVB1.2); protein 5 (1_D08_A8_4_DVB1); protein 6 (1_H10_F10_DVA2-1); protein 15 (1_G01_E8_3_DVB2); protein 45 (Consensus_41); protein 66 (1_G11_B10_3_PM). Based on similarity with known toxins in the structural family, the presumed biological function of in *D. quadriceps* venom OBP/PBP-like protein, appears to be of an extremely potent allergy-inducing agent, that causes IgE antibody production.

This protein was identified from the SDS-PAGE gel bands 10, 11, and 12 (with peptide fragments covering 18% of the full sequence) and from the in-solution proteomic analysis protein 3 (with peptide fragments covering 32% and 31% of the full sequence). It corresponded to the protein product of the *D. quadriceps* venom gland transcript Contig8_B2-2, Figure S7) that codes for the major ant venom allergen 2/4-like (Odorant/pheromone-binding protein-, OBP/PBP-like). This identified toxin also corresponds to the predicted gene product of a gene segment of the *D. quadriceps* genome [XP_014486970.1]. The theoretical molecular mass predicted for the mature sequence of this 119-aa long peptide is 12,984.28 Da. In Figure 8, the alignment of Venom Allergen 2/4-like (OBP/PBP-like) from the *D. quadriceps* venom and the venom proteins Sol i 2 and Sol i 4 from *S. invicta* is shown.

#### 2.2.8. *Dinoponera quadriceps* Bovine Pancreatic Trypsin Inhibitor (BPTI)/Kunitz-Like Serine Protease Inhibitor

**Figure 9:**
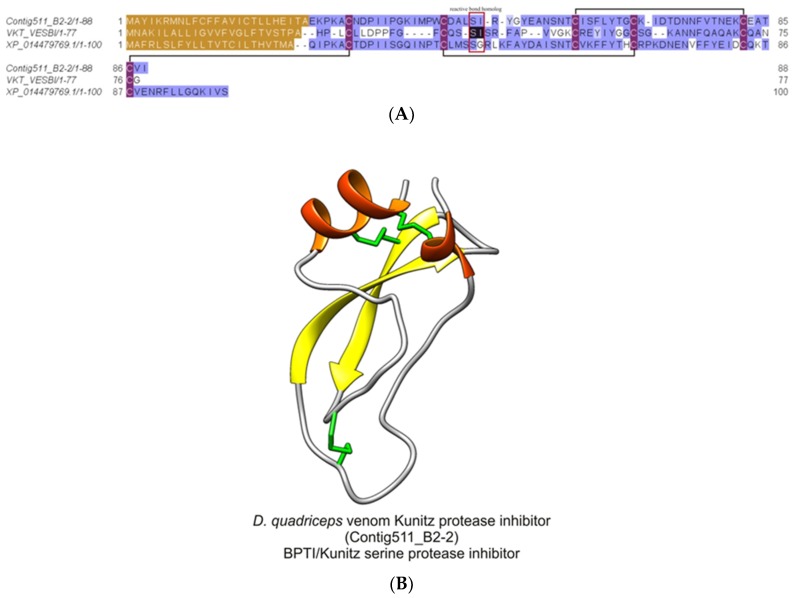
Structural analysis of *D. quadriceps* BPTI/Kunitz-type peptide toxin. (**A**) *D quadriceps* venom BPTI/Kunitz-type peptide was aligned with the bicolin peptide (Kunitz-type serine protease inhibitor bicolin) from black shield wasp *V. bicolor* venom (VKT_VESB) and with a predicted sequence identified in gene segment of *D. quadriceps* genome, the Kunitz-type serine protease inhibitor ki-VN-like (XP_014479769.1). The amino acid residues that located at the inhibitory active site, in bicolin peptide, are indicated by red box and are shown in black, for comparison. The connectivity pattern of the three disulfide bonds are indicated by solid black lines. The signal peptide is shown in brown. Sequence similarity was determined using BLOSUM62. (**B**) the structural model of *D. quadriceps* Kunitz-type toxin predicted from the venom gland transcript Contig511_B2-2, identified by in-solution proteomics (e.g., protein 39, Table S2) was elaborated by homology using as template the mambaquaretin-1 toxin (PDB 5M4V), a selective antagonist of the vasopressin type 2 receptor (V2R) from the green mamba *Dendroaspis angusticeps* venom. Mambaquaretin-1 is an efficient antagonist of the V2R activation pathways that involve cAMP production, beta-arrestin interaction, and MAP kinase activity [30], thus the presumed biological function of *D. quadriceps* Kunitz-type peptide in the venom. The typical structure is characterized by an α/β protein with few secondary structures that is constrained by 3 disulfide bonds.

From the in-solution-based proteomics analysis, this *D. quadriceps* venom component corresponding to the protein product of the transcript contig511_B2-2 (protein 39) was identified (Table S2), and its structural organization can be seen in Figure S8. The mature protein, a bovine pancreatic trypsin inhibitor (BPTI) /Kunitz-like toxin, is stabilized by three disulfide bonds, as shown by the alignment of *D. quadriceps* BPTI/Kunitz-like toxin with its homologue in wasp and with another sequence predicted from the giant ant genome segment (Figure 9).

#### 2.2.9. Pilosulin- and Ponericin-Like Peptides

**Figure 10:**
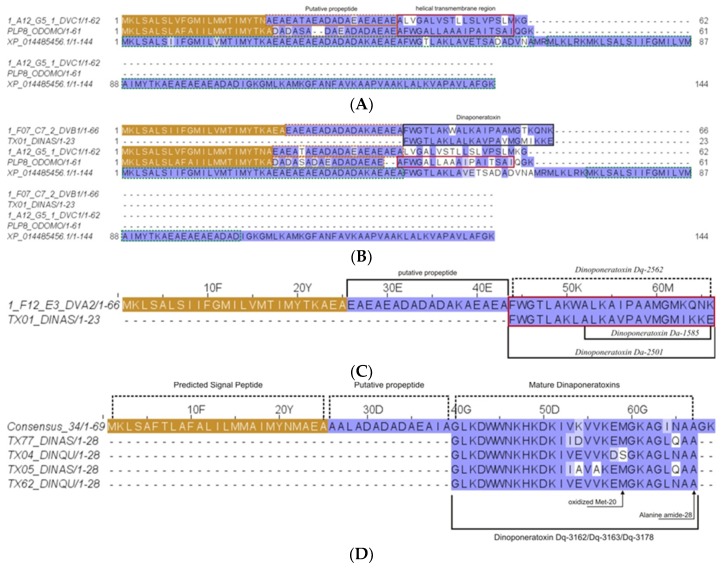
Dinoponeratoxins (DNTxs), pilosulin- and ponerecin- like peptides found by proteomic and transcriptomic analysis as part of the major components of *D. quadriceps* venom. (**A**) Sequence that corresponds to mature peptide encoded in the transcript contig 1_A12_G5_1_DVC1, a pilosulin-like precursor peptide [Dq-1969]; (**B**) Contig 1_F07_C7_2_DVB1, a pilosulin-like precursor peptide [Dq- 2532]; (**C**) product from transcript Contig Consensus 34, a pilosulin-like precursor peptide; (**D**) product from transcript contig 1_F12_E3_DVA2, a pilosulin-like precursor peptide [Dq-2562]. The peptide signal in each case is colored in gold, the prepropeptide in magenta. The mature, processed peptide and fragments are boxed in red and black and the experimental molecular mass indicated. Note. the *D. quadriceps* dinoponeratoxins, pilosulin- and ponerecin- like peptides detected in the venom proteome are listed in Table S2.

From the in-solution-based proteomics analysis, four dinoponeratoxins (DNTxs) corresponding to contigs 1_A12_G5_1_DVC1 (protein 26), 1_F07_C7_2_DVB1 (protein 22), consensus 34 (protein 21), and 1_F12_E3_DVA2 (protein 46) in the *D. quadriceps* venom gland transcriptome were identified (Table S2). The transcript precursors of these DNTxs, with their respective peptide products, are shown in Figure S9A–D. In Figure 10, the complete amino acid sequences are shown as aligned with their recognized homologues from the *Dinoponera* genus and other species of hymenopterans.

#### 2.2.10. Inhibitor Cysteine Knot (ICK)-Like Venom-Peptides

**Figure 11:**
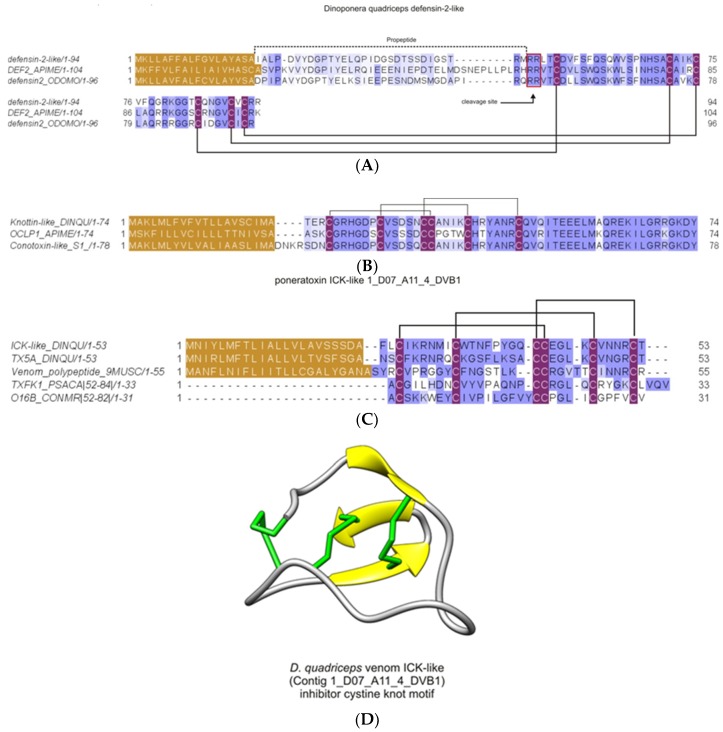
Knottins, ICK-like toxins, found in the crude venom of the giant ant *D. quadriceps*. (**A**) Defensin-like-2 venom-peptides that corresponds to the gene product of the nucleotide sequence XM_014620749.1 from *D. quadriceps*. (**B**) Knottin-like toxin found as the product from the venom gland transcript contig516_B2-2 gi|578895399|. (**C**) Sequence that corresponds to mature homologous peptide encoded in the transcript clone 1_D07_A11_4_DVB1 (U1-poneritoxin-Dq5a) from *D. quadriceps* transcriptome. The peptide signal in each case is colored in gold, the prepropeptide is indicated by a dashed line (in A). Disulfide bonds are represented by connecting solid lines, as known from the S-S patterns of homologous sequences. The mature, processed toxins are seen downstream the cleavage site, in the prepropeptide (in A) or downstream the signal peptides (in B and C). The gene and protein database access codes are as follow: DEF2_APIME [Q5MQL3], defensin-2 from the honey bee *A. mellifera*; A0A348G5W3; Conotoxin-like_S1 from the ponerine ant *O. monticola*; A0A348G6A9_ODOMO, defensin2_ODOMO: defensin 2 from *O. monticola*; OCLP1_APIME [H9KQJ7], omega-conotoxin-like protein 1 de *A. mellifera*; A0A3G5BID7_9MUSC, venom polypeptide from the giant assassin fly *Dolopus genitalis*; O16B_CONMR [Q26443], Na^+^-sodium channel gating-modifier toxin ω-conotoxin MrVIB from the sea snail *Conus marmoreus*; TXFK1_PSACA U1-theraphotoxin-Pc1a [P0C201] from the spider *Psalmopoeus cambridgei*; (**D**). Structural model of *D. quadriceps* ICK-like venom peptide predicted from the venom gland transcript contig1_D07_A11_4_DVB1. The structural model was predicted by homology modelling, using as template the toxin U5- scytotoxin-Sth1a (PDB 5FZX) from the venom of the Spitting Spider *Scytodes thoracica*. The function is still elusive, despite the potentiality to modulate ion-channel activity and neural receptors. This structure displays the classical short triple-stranded antiparallel beta-sheet of knottins, short peptides.

From the in-solution proteomics analysis, three types of knottins (1_D07_A11_4_DVB1-protein 88; Contig516_B2-2 gi|578895399| - protein 77; XM_014620749.1 - protein 99), referred to as ICK-like toxins, were found in the venom of *D. quadriceps* (Figure 11). The experimental data are presented in summarized information in Table S2. The transcript precursors for two of them are shown in Figure S10A,B. These ICK-like toxins (Figure 11A,B) expressed in the giant ant venom are consistent with similar structures that are known neurotoxins in other venoms, like spider huwentoxins and anemone ICKs (e.g., BcsTx3 and κ-actitoxin-Bcs4a), while the third ICK-like toxin is structurally similar to defensin-like-2 of insects (hymenopteran canonical invertebrate defensin-like-2 peptides). In Figure 11A, the *D*. *quadriceps* ICK-like toxins that share high level of similarity (~60–90%) with honey bee and spider homologues are compared by amino acid sequence alignment. In Figure 11B, the giant ant knottin-like peptides that are highly similar (>90%) to conotoxin-like neurotoxins from *A. mellifera* and *Conus* sea snail are presented. The defensin-like venom peptides from *A. mellifera* and *Odontomachus monticola*, with antimicrobial activity associated to their structures, are shown in C, and are compared with similar (~50% identical) sequence found in the venom of *D. quadriceps*.

## 3. Discussion

Bottom-up proteomics strategies have been applied to study the venom protein profiles of several animals, such as cnidarians [31], mollusks [32], snakes [33], and ants. In fact, the compositional differences of the bullet ant *P. clavata* crude venoms that were collected by manual gland dissection and electrical stimulation were thus elucidated [34]. In another study, the venom protein content of the ponerine ant *Pachycondyla striata* was determined employing both in-gel (2-D electrophoresis) and in-solution digestion proteomics, resulting in the identification of 42 spots and 5 proteins [35]. Recently, several proteins were identified from the venom of the trap jaw ant *O. monticola* by in-solution digestion venom proteomics [36].

In the present study, the experimental data of our venomic analysis, which was performed using both in-gel and in-solution-based proteomics approaches, was compared with two transcriptomic datasets of the *D. quadriceps* venom glands, namely, (1) the predicted peptide products of 496 contigs from 800 Applied Biosystems (ABI) Prism files from the Sanger sequencing of venom gland cDNA library and (2) 18,546 assembled transcripts from more than 2,500,000 raw reads obtained from Ion Torrent RNA sequencing [23]. The majority of proteins identified by gel-based proteomics confirmed the transcriptional repertories expressed in the *D. quadriceps* venom gland and counterparts found in the venoms of not only other ant species, but also of wasps and honey bee, despite of their species-specific composition. For instance, in the *D. quadriceps* venom proteome, there are toxins that shared similarity with the venom dipeptidyl peptidase-4 from the parasitic ant *Ectatomma tuberculatum* [37]; the odorant-binding protein and venom allergen 2 from the fire ant *S. invicta* [7]; the phospholipase A1 2, hyaluronidase, venom allergen 3, and glucose dehydrogenase FAD from the stinging ant *P. striata* [35]; and the hyaluronidase and venom allergen 2 from the ponerine ant *O. monticola* were identfied [36]. Moreover, the intra- and interspecific variation of venom components is a known biological phenomenon observed from arthropods to snakes [38,39,40] herein observed.

The limit of detection for proteins in the Coomassie-stained polyacrylamide gels is approximately 50 ng with a dynamic range of up to 500 ng. Based on this, the resolution of the giant ant crude venom with one-dimensional SDS-PAGE allowed the visualization of the predominant venom polypeptide components of *D. quadriceps* (Figure 1, Table S1). Accordingly, the venom of the giant ant *D. quadriceps* is predominantly composed of venom allergens (major venom allergen 3 and 2/4-like), hydrolases (dipeptidyl peptidase-4, hyaluronidase, GDH, and PLA1), major royal jelly-like protein, and Kunitz-type toxin. However, considering the data from the in-solution proteomics, two additional classes of toxins of lower molecular mass should be included in the giant ant venom composition: pilosulin- and ponericin-like peptides (dinoponeratoxins) and cysteine-stabilized knottins (ICK-like). These experimental data are summarized in Tables S1 and S2.

In the venom cocktail, these polypeptide components can act in combination to efficaciously exert their effect in interfering with victim and/or prey homeostasis. For instance, the venom dipeptidyl peptidase-4-like can modulate the chemotactic activity of immune cells after the sting and trigger immunoglobulin E (IgE)-mediated allergic reactions produced by basophils. Such venom enzyme is also known as allergen C (Api m 5) in the honey bee *A. mellifera* and Ves v 3 in the common wasp *V. vulgaris*. It is also known as the major allergen of the European paper wasp *Polistes dominula* venom [28,41]. However, the role of glucose dehydrogenase [FAD, quinone] in the venom and venom gland of *D. quadriceps* remains unknown. In fruit fly *D. melanogaster*, similar enzyme, UniProtKB–P18173 (DHGL_DROME), is responsible for cuticular modification during morphogenesis. Notably, the homologues of glucose dehydrogenase [FAD, quinone] have being identified in the venom proteome of the parasitic ant *E. tuberculatum* [42], although it was not described as a genuine insect antigen or venom component.

The MRJPs sequence identified in the transcriptome and venom proteome of *D. quadriceps* might represent a new member of the same family, but with distinct function from its counterparts in bee and fruit fly. The MRJPs is known to have anti-apoptotic activity and act in similar way that the nerve growth factor (NGF) does in hepatocyte culture, interacting with mitogen-activated protein kinase and protein kinase B, controlling the pattern of adult fly cuticle pigmentation and larval buccal parts [43]. It is important to note that the MRJP-like protein of *D. quadriceps* shares only 27% of similarity with the *A. mellifera* MRJP-1 and 40% with the *D. melanogaster* yellow protein, which controls the pattern of pigmentation of the fly cuticle and larvae. The yellow proteins (MRJPs) are involved in the enzymatic conversion processes of the melanization pathways [44]. It is interesting note that the C-terminal end of the bee MRJP-1 can be cleaved in jellein-2, which is subsequently cleaved in jellein-1 and jellein-4, which in turn show antimicrobial activity [45]. The jellein-related peptides were not observed in the *D. quadriceps* venom either because the *D. quadriceps* MRJP-1 was not cleaved or because a molecular weight cut-off of 10 kDa was defined for polypeptide analysis in this study. Indeed, it would be necessary to verify if the C-terminus of the *D. quadriceps* MRJP-1 could be cleaved into smaller peptides, similar to jellein antimicrobial peptides, since the *D. quadriceps* MRJP-like venom protein displays a C-terminal segment that has alternating hydrophilic and hydrophobic residues and is rich in proline residues (see green box in Figure 5), close to the lysine residues, the likely site of proteolytic cleavage.

The phospholipases A2 are the main components of bee venoms, while PLA1s are found as the main allergens in wasp venom (e.g., Sol i1, Api m1, Bom p1, Bom t1, Ves v1, Ves m2, Ves s1, Vez c1, Pol a1 and Pol e1). Based on the structural similarity and conservation, the *D. quadriceps* venom PLA1 could cause hemorrhagic disorders and exacerbate envenoming response as a result of its expression in the venom and catalytic activity that generates pharmacologically active lipids. The wasp PLA1 catalyzes the hydrolysis of emulsified phospholipids exclusively at the sn-1 position, releasing free fatty acids and lysophospholipids. Lysophospholipis are multifunctional mediators and second messengers involved in diverse cellular processes, including platelet activity and blood coagulation, as well as in the physiopathology of a number of human diseases [46]. The phospholipase A1 from the black-bellied hornet wasp (*Vespa basalis*) was shown to display a potent hemolytic activity that is responsible for their lethal effects [47,48]. The roles of wasp venom PLAs in platelet activation and allergic response have been reported [49,50]. The roles of PLA1 and PLA2 in the envenomation and allergic response caused by wasps, as well as the involvement of venom PLA1 in allergy caused by ant stings, is reviewed elsewhere and further information can be taken [26,51]. In this context, the PLA1 from giant ant venom is not only an enzymatic component (hydrolase), but also appears as a potential allergen that contributes to trigger a late immune response in the victim.

In the venom of *D. quadriceps*, enzymes are one of the main components that apart from their role in catalytic destruction of tissues that can contribute to allergic responses. Among these venom-enzymes, the hyaluronidase-type found in the *D. quadriceps* crude venom shares structural characteristics with the wasp hyaluronidase; for example, two intra-molecular disulfide bridges formed between four cysteine residues located in conserved positions in contrast to the mammalian hyaluronidases that have four disulfide bonds. The wasp and honey bee hyaluronidases are known allergens found in the hymenopteran venoms (e.g., Ves v2 and Api m2) and, in their catalytically active forms, additionally appear to contribute, together with phospholipases, to the facilitation of venom dissemination [52,53]. In this respect, the venom of the giant ant *D. quadriceps* is comparatively more similar to the venom of wasps and other unrelated ants, like the red fire ant *S. invicta*.

It is well known that allergy is one of the main systemic symptoms in humans injected with hymenopteran venoms. Two other classes of allergens detected in the *D. quadriceps* venom are polypeptides highly similar to the major venom allergen 3 and major ant allergen 2/4-like that we will discuss in the following lines. The venom allergen 3 is the homologue of the red fire ant *S. invicta* Sol i 3 (major venom allergen 3/cysteine-rich venom protein–CAP/SCP superfamily; cysteine-rich secretary protein (CRISP) family). Proteins of this family are characterized by high structural conservation, especially in the arrangement of their disulfide bridges in the CRISP domain, but with high diversity beyond this central region that dramatically alters the target specificity and, therefore, the range of biological activities. Some proteins of the SCP/CAP family have a small CRISP domain, which is found in mammalian proteins and snake toxins, by which they were shown to regulate calcium signaling via the ryanodine receptor [54]. The Sol i 3 antigen is the most common venom polypeptide responsible for allergic hypersensitivity caused by insects in the southeastern United States [55]; therefore, the major venom allergen 3/cysteine-rich protein-like venom component in *D. quadriceps* could play a critical role to trigger allergy. Interestingly, in humans, the CRISP family proteins are most often secreted and have an extracellular endocrine or paracrine function; are involved in processes that include the regulation of matrix morphogenesis and extracellular branching, inhibition of proteases, ion-channel regulation and cell-cell adhesion in fertilization; and act as tumor suppressor or promoters in tissues, including the prostate.

The other venom allergen found preponderantly in the *D. quadriceps* venom belongs to a family of carrier proteins similar to insect pheromone/odorant-binding proteins that is homologous to the main antigen in the fire ant *S. invicta* (Sol i 2) venom (ant venom allergen 2/4 family). Sol i 2 and the related Sol i 4 are potent antigens of the red fire ant venom capable of triggering anaphylaxis [8,56]. The antigen Sol i 2 causes the production of IgE antibodies in approximately one-third of the individuals stung by fire ants. Based on the structural characteristics of Sol i 2 and Sol i 4, they can form homo and/or heterodimers. The recombinant dimeric Sol i 2 produced in baculovirus was crystallized as a native and seleno-methionylated derivative protein, and its structure was determined at 2.6 Å resolution. Its structure is stabilized by three intramolecular and one intermolecular disulfide bridges [57]. Conceivably, Sol i 2 might play a biological role in capturing and/or transport small hydrophobic ligands, such as pheromone, odorant molecules, fatty acids, or short-lived hydrophobic messengers. The venom allergen 2/4-like protein in *D. quadriceps*, herein identified and encoded in the venom gland transcript contig8_B2-2, has one less disulfide bridge in contrast to the *S. invicta* counterpart, which might be considered and grouped with *D. quadriceps* venom allergen 2/4-like polypeptide as a new member of the family of insect pheromone/odorant-binding proteins in hymenopterans. Moreover, based on the fragments detected from the in-gel proteomics analysis, at least three isoforms of similar proteins belonging to the ant venom allergen 2/4 family are expressed in the *D. quadriceps* venom. Altogether, the diverse classes of venom allergens comprise a significant part of venom components in *D. quadriceps* that could effectively cause local and systemic hypersensitivity and immuno-reaction in human and small mammals, being useful to avoid aggressors and predators.

Another important group of toxins in the *D. quadriceps* venom are DNTxs, which are pilosulin- and ponerecin-like peptides that could be classified into short- and long-DNTxs, ranging in size from 11 to 28 residues. They are present in large proportion in the venom gland transcriptome and venom proteome and display membranolytic, cytolytic, antimicrobial, and antiparasitic activities [10,24,25,58]. Given that DNTxs comprise a predominant fraction of transcripts expressed in the *D. quadriceps* venom gland, long and short DNTxs were synthesized and evaluated for their anti-infective activity. Against trypanosomatid parasites (i.e., *Trypanosoma cruzi*), they displayed variable levels of effect, but good profiles of anti-trypanosome activity, better than the drug-of-choice [58]. The *D. quadriceps* DNTxs share structural similarities with antimicrobial peptides, such as ponericins G2/G3 from ponerine and poneromorph ants, and frog temporins Brevinin-1PTa and Gaegurin-5, in case of short-DNTxs, and frog dermaseptin-H65 (and ant ponericins W3/W5, Q49/Q50), in case of long-DNTxs, from vertebrates [10,23,24,25]. In the present study, four types of DNTxs were found in the in-solution, but not the in-gel proteomics analysis, because of the exclusion size and cut-off value of each technique that selected peptide fragments for analysis. Despite of this fact, DNTxs are the components that contribute to the potency of the venom and, in combination with allergens, play a role in defense and predation.

Last but not the least, cysteine-stabilized peptides, like inhibitor cystine-knot (knottin, ICK-like) peptides are relatively rare in ant venoms, with few examples reported to date [9]. One of these examples is the ICK-like toxins (knottins) annotated from the venom gland transcriptome of *D. quadriceps*. Here, in the *D. quadriceps* crude venom proteomics, three homologous (two of which correspond to the expressed transcript products) were identified through the analytical procedure #2 of peptides generated by in-solution digestion. Found in low abundance, as compared with other venom toxin components, the ICK-like toxins in the *D. quadriceps* venom contrast with the predominance of this class of toxins in spider venom, where dozens of peptides with insecticidal roles are found and grouped in diverse families [59]. Spider ICK-like toxins act mainly on the voltage-gated sodium channels that usually involve three different mechanisms: inhibition of channel opening, prevention of fast inactivation, and facilitation of channel opening, but the final pharmacological effect is the disruption of normal neurotransmission [60]. The other knottin (ICK)-like peptide found in the venom of *D. quadriceps* shares similarity with defensin-2 of insects (Figure 11). Invertebrate (insect) defensins comprise a family of cysteine-stabilized antimicrobial peptides, primarily active against the Gram-positive bacteria. These defensins have been found in arthropods (insects, ticks, spiders, and scorpions), in bivalve mollusks, and even in fungi, but are unrelated to mammalian defensins [61]. The low predominance of the ICK-like toxins in the venom of *D. quadriceps* and in ants, in general, might be the result of the evolutionary pressure and the efficiency of linear and cytolytic peptides, as well as hydrolases (e.g., phospholipases), in prey paralysis and (2) the potency of ICK-like neurotoxins that might be sufficiently high to achieve an efficacious paralyzing effect. Interestingly, the venoms of ancient marine organisms, like cnidarians, are rich in cytolysins and phospholipases that constitute numerous neurotoxins that cause paralysis for prey hunting [62,63]. Taking these facts into account, the biological activity of the ICK-like toxins in *D. quadriceps* should be further investigated, given that they are potentially highly selective ion-channel disruptors, and consequently presumable, insecticidal and germicidal.

Based on our proteomic data, the combined effect of paralysis to hunt prey and induction of allergenicity, to avoid aggressors and predators, seem to be consistent with the compositional content of the *D. quadriceps* venom. Moreover, the cytolytic and membranolytic predicted properties of giant ant toxins to disrupt cell membranes and tissues, presumably contribute to toxin diffusion by increasing blood vessel permeability, thereby facilitating the spread of neurotoxins and allergens and, thus, provoking allergic reaction and hypersensitivity. It is interesting to note that in wasps, the predominance of venom allergens and (hydrolytic) enzymes as components of a toxin cocktail or the high content of neurotoxic peptides and disruptors of primary metabolism in other kind of venom mixture is associated with the behavior pattern of a given wasp species, that is, if a given wasp species is social or solitary. Solitary wasps are eminent hunters that immobilize prey with neurotoxins and metabolic disruptors for oviposition, while social wasps defend their colonies for eventual threats with allergens. Thus, the solitary wasp venom contains toxins that cause paralysis and restrain the metabolism for a very basal status, while the social wasp venom recruits toxins that cause pain and strong allergic reactions for defense [52]. In this view, based on our study, *D. quadriceps* appears to have both paralyzing toxins for hunting and allergens to defer attacks by virtue of their small colonies, with no more than hundreds of individuals [64]. Thus, the composition of the *D. quadriceps* venom, as seen here, seems to reflect a dual role: a toxin cocktail for defense and attack, from the ecological point of view, and a composition of venom polypeptides that cause long-lasting pain and systemic symptoms, from the medical aspects of human envenoming and accidents with (“false”) tucandiras.

## 4. Conclusions

Using the bottom-up proteomic approach, we investigated the protein content of the giant ant *D. quadriceps* crude venom. We identified predominant components that are venom enzymes, allergens, and cytolytic and accessory polypeptides that together appear to potentially promote the diffusion of toxins and trigger allergic responses in prey and victims. Moreover, the less abundant ICK-like toxins could contribute to the paralyzing effect of this predatory giant ant species (*D. quadriceps*). Overall, the present work, shed light on the description of the venom composition of one of the thousands of species of ants and hymenopterans that inhabit Earth.

## 5. Materials and Methods

### 5.1. Ant Sampling and Venom Extraction

Adult *D. quadriceps* individuals (~100) were collected as described in one of our previous report [23] and maintained in a terrarium. For venom extraction, these 100 individuals were individually immobilized with a flexible clamp, and venom was collected using a capillary tube that was positioned in the back of an ant’s gaster. After extraction, the venom was immediately transferred to ice and pooled. The pool of crude venom was then frozen in liquid nitrogen, lyophilized and stored at −20°C until required for proteomic analysis. This procedure was repeated every two weeks. The access for sampling and studying the venom content of *D. quadriceps* was registered under the numeric codes 28794-1 and A1D1ACF in the System of Management of Genetic Heritage and Associated Traditional Knowledge (SISGEN), Ministry of Environment, Federal Government of Brazil.

### 5.2. Proteomic Analysis

#### 5.2.1. Separation of Venom Protein by Denaturing Polyacrylamide Gel Electrophoresis (PAGE)

The *D. quadriceps* crude venom was resolved by protein electrophoresis according to Laemmli [65]. The dried venom (30 µg) was solubilized in sample buffer and separated by sodium dodecyl sulfate-polyacrylamide gel electrophoresis (SDS-PAGE, 12.5% T, 2.8% C), under reducing conditions. Protein bands were visualized with Coomassie blue, using a rapid gel staining protocol. A low-molecular mass protein calibration kit for electrophoresis (Amersham, GE Healthcare, Chicago-IL, USA) was employed in this study.

#### 5.2.2. In Gel Digestion and Mass Spectrometry Analyze

The in-gel digestion was conducted according to Westermeier, Naven, and Höpker [66] with small modifications. Firstly, the gel bands were selected, excised, and transferred to a 1.5-mL micro tube. Subsequently, a solution of 75 mM ammonium bicarbonate (in 40% ethanol) was added to destain the bands. Thereafter, the supernatant was removed, 5 mM dithiothreitol (in 25 mM ammonium bicarbonate) was added, and all samples were incubated at 60 °C for 30 min (reduction step); next, we added 55 mM iodoacetamide (in 25 mM ammonium bicarbonate) and incubated all samples at room temperature for 30 min in the absence of light. The supernatant of all individual samples was again removed, and the gel pieces dehydrated by adding acetonitrile (ACN). Subsequently, to each sample, 10 µL of proteomic grade trypsin solution (10 ng/µL in 50 mM ammonium bicarbonate) was added, and digestion was allowed for 45 min on ice. Thereafter, supernatants were removed, gel pieces were covered with 50 mM ammonium bicarbonate and incubated overnight at 30 °C. Finally, each sample was suspended in 20 µL of ACN/5% trifluoroacetic acid (TFA) (1:1, *v*/*v*) and sonicated for 10 min. The supernatant was again removed and dispensed in a separate tube. We repeated this step three times and combined the supernatants of the same samples. Lastly, we repeated the process using ACN instead of ACN/5% TFA. The obtained supernatant was combined with the previously obtained supernatants.

The tryptic peptides were analyzed by liquid chromatography–mass spectrometry (LC–MS) using an electrospray-ion trap-time of flight (ESI-IT-TOF) system coupled to a binary ultra-fast liquid chromatography system (UFLC) (20A Prominence, ShimadzuKyoto, Japan).. Briefly, samples were dried, resuspended in 0.1% acetic acid, and loaded onto a C18 column (Discovery C18, 5 μm, 50 × 2.1 mm) operating with a binary solvent system: (A) water:acetic acid (999:1, *v*/*v*) and (B) ACN:water:acetic acid (900:99:1, *v*/*v*/*v*). The column was eluted at a constant flow rate of 0.2 mL/min with a 0 to 40% linear gradient of solvent B for 40 min. The eluates were monitored by a Shimadzu SPD-M20A PDA detector before introduction into the mass spectrometer. The interface voltage was set to 4.5 KV, the capillary voltage used was 1.8 KV at 200 °C, and the fragmentation was induced by argon collision at 50% ‘energy’. The MS spectra were acquired under the positive mode and collected in the range of 350 to 1400 *m*/*z*. The MS/MS spectra were collected in the range of 50 to 1950 *m*/*z*.

#### 5.2.3. In-Solution Digestion and Mass Spectrometry Analysis

##### Analysis #1

One milligram of the *D. quadriceps* venom was solubilized in 1.0 mL of 0.1% formic acid, and a-250 µL aliquot was concentrated using a 10-kDa cut-off centrifugal filter (Amicon^®^ Ultra-4 Centrifugal Filter Unit, Sigma-Aldrich, St. Louis-MO, USA). The retentate (peptide fraction >10 kDa) was dried and submitted to in-solution digestion according to Beraldo Neto and colleagues [67]. Briefly, the venom protein samples in 8 M urea were reduced using TCEP-HCl (20 mM Tris(2-carboxyethyl)phosphine hydrochloride, TCEP) at room temperature (RT) for 1 h and then alkylated (10 mM iodoacetamide, IAA) at RT for 1 h in the absence of light. Thereafter, the samples were diluted to a urea concentration of <2 M (with 100 mM Tris-HCl, pH 8.5), followed by the addition of 10 µL proteomic-grade trypsin (10 ng/µL in 100 mM Tris-HCl, pH 8.5). The incubation was performed at 30 °C overnight, and the enzymatic reaction was stopped by adding 50% ACN/5% TFA. The sample was lyophilized, desalted, and concentrated using a ZipTip^®^ C-18 pipette tips (Millipore Co., Burlington, MA, USA). We repeated this step twice, pooling the material of the same samples and drying.

Finally, we resuspended the dried material in 5 μL of 0.1% formic acid, and 1 μL was automatically injected in the EASY Nano LCII system (Thermo Fisher Scientific) into the top 5 cm of a 10-μm Jupiter C-18 trap column (100 μm I.D. × 360 μm O.D.) coupled to an LTQ-Orbitrap Velos mass spectrometer (Thermo Fisher Scientific, Waltham,-MA, USA). The chromatographic separation was performed on a 15-cm long column (75 μm I.D. × 360 μm O.D.) packed in-house with 3-μm ReproSil-Pur C-18 beads (Dr. Maisch GmbH, Ammerbuch-Entringen, Germany) in a binary system: (A) water:formic acid (999:1, *v*/*v*) and (B) ACN:formic acid (999:1, *v*/*v*). The column was eluted at a constant flow rate of 300 nL min^−1^ with a 5% to 35% linear gradient of solvent B for 75 min. The spray voltage was set at 2.2 kV, and the mass spectrometer was operated in the data-dependent mode, in which one full MS scan was acquired in the *m*/*z* range of 300–1,600, followed by an MS/MS acquisition using the collision-induced dissociation of the ten most intense ions from the MS scan. The MS spectra were acquired in the Orbitrap analyzer at 30,000 resolution (at 400 *m*/*z*), whereas the MS/MS scans were acquired in the linear ion trap. The minimum signal threshold to trigger the fragmentation event, isolation window, activation time, and normalized collision energy were set to 1000 cps, 2 *m*/*z*, 10 ms, and 35%, respectively. We applied a dynamic peak exclusion list to avoid the same *m*/*z* of being selected for the next 20 s.

##### Analysis #2

One hundred micrograms of the *D. quadriceps* venom was analyzed by in-solution digestion, as described in the previous section. The solution of tryptic-digested venom polypeptides was lyophilized, desalted, and concentrated using a ZipTip^®^ C-18 pipette tips (Millipore, Co., Burlington, MA, USA). This step was repeated twice, and the resulting peptide solutions were pooled and dried. Finally, the dried tryptic peptides were solubilized with 5 μL of 0.1% formic acid, and 1 μL was automatically injected into a 15 cm × 50 µm Acclaim PepMap™ C-18 column (Thermo Fisher Scientific, Waltham,-MA, USA) assembled in a nano chromatographer (EASY-nLC 1200 system, Thermo Fisher Scientific, Waltham,-MA, USA) coupled to a Q Exactive Plus mass spectrometer (Thermo Fisher Scientific, Waltham,-MA, USA). A binary solvent system was used: (A) water:formic acid (999:1, *v*/*v*) and (B) ACN:water:formic acid (800:199:1, *v*/*v*/*v*). The peptides were eluted at a constant flow rate of 300 nL/min with a 4% to 40% linear gradient of solvent B for 100 min. Spray voltage was set at 2.5 kV, and the mass spectrometer was operated in the data-dependent mode, in which one full MS scan was acquired in the *m*/*z* range of 300–1,500 followed by MS/MS acquisition using the higher-energy collision dissociation (HCD) of the 10 most intense ions from the MS scan. The MS and MS/MS spectra were acquired in the Orbitrap analyzer at 70,000 and 17,500 resolutions (at 200 *m*/*z*), respectively. The maximum injection time and automatic gain control (AGC) target were set to 25 ms and 3 × 10^6^ for a full MS, and 40 ms and 105 for MS/MS, respectively. The minimum signal threshold to trigger fragmentation event, isolation window, and normalized collision energy (NCE) were set to 2.5 × 10^4^ cps, 1.4 *m*/*z* and 28, respectively. A dynamic peak exclusion was applied to avoid the same *m*/*z* of being selected for the next 30 s.

### 5.3. Data Processing and Data Analysis

#### 5.3.1. In-Gel Digestion

The LCD Shimadzu raw data were converted to the mascot generic format (MGF) files with the software LCMS Protein Postrun (Shimadzu, Kyoto, Japan) and loaded in the software Peaks Studio V7.0 (Bioinformatics Solutions Inc, BSI, Waterloo-ON, Canada) [68]. Proteomic identification was performed according to the following parameters: error mass (MS and MS/MS) set to 0.1 Da; methionine oxidation and carbamidomethylation were set as variable and fixed modifications, respectively; trypsin as proteolytic enzyme for cleavage; maximum missed cleavages (3), maximum variable PTMs per peptide (3), and non-specific cleavage (one); the false discovery rate was adjusted to ≤1% and only the proteins with score ≥30 and containing at least 1 unique peptide were considered in this study. All data were analyzed against a *D. quadriceps* transcriptomic database (6510 entries; National Center for Biotechnological Information (NCBI) BioProject: PRJNA217939) [23], compiled in 17 April 2016.

#### 5.3.2. In-Solution Digestion

RAW files were directly loaded in the software Peaks Studio V7.0. The following parameters were specifically adjusted; for analysis #1, the MS and MS/MS error mass were set to 15 ppm and 0.5 Da, respectively and for analysis #2, the MS and MS/MS error mass were set to 10 ppm and 0.01 Da, respectively. The following parameters were used in both analyses: methionine oxidation and carbamidomethylation were set as variable and fixed modifications, respectively; trypsin as cleavage enzyme; maximum missed cleavages (3), maximum variable PTMs per peptide (3), and non-specific cleavage (both); the false discovery rate was adjusted to ≤0.1%; only the proteins with score ≥50 were considered in this study. We analyzed all data against a *D. quadriceps* transcriptomic database (29909 entries; built by downloading and merging two transcriptome databases: NCBI BioProjectsPRJNA301625 [69] and PRJNA217939 [23], compiled in 6 August 2018. 

After analysis with the Peaks software, the in-gel and in-solution proteomics data were interpreted according to the following rationale:

(a) only one protein for each group (each group containing the proteins identified by a common set of peptides was maintained—Peaks software classification);
(b) If one group contained more than one identified protein, the first protein was maintained and the other protein hits were considered redundant and, thus, were removed;
(c) Despite the item b, additional 9 groups were left aside because they contained redundant sequences (three groups from the “in-solution analysis (i)” data and 6 groups from the “in-solution analysis (ii)” data);
(d) both in-solution datasets and highlighted proteins containing the same contig names were compared;

Based on this workflow, a basic local alignment search for proteins (BLASTx) was performed with all identified sequences, limiting the search for the Hymenoptera order (taxid: 7399). Thus, an alignment with a higher score was achieved in this study.

### 5.4. Structural Models of Selected Dinoponera Quadriceps Venom Toxin

The homology models of selected toxins were elaborated by means of the Swiss-model server [70]. Molecular graphics and analyses were performed with the UCSF Chimera package, developed by the Resource for Biocomputing, Visualization, and Informatics at the University of California, San Francisco (supported by NIGMS P41-GM103311). In all secondary structure models in this study the α-helix is highlighted in red, β-sheets in yellow, coil/turns in gray, and disulfide bounds in green.

## Supplementary Materials

The following are available online at https://www.mdpi.com/2072-6651/11/8/448/s1: Figures S1–S10: Structural organization of toxin precursor transcripts that matched identifiers in proteomic analysis. Tables S1 and S2: Lists of peptide toxins found in the venom of giant ant *D. quadriceps* identified by in-gel and in-solution proteomic analysis, respectively.

## Abbreviations

BPTI	Bovine Pancreatic Trypsin Inhibitor
CRISP	Cysteine-rich secretory proteins
ICK	Inhibitor Cysteine Knott (Knottin)
LC-ESI-MS	Liquid chromatography-electrospray ionization mass spectrometry
MRJP	Major royal jelly protein
PDB	Protein Data Bank
PLA1	Phospholipase 1
PLA2	Phospholipase 2
SCP	SCP-like extracellular protein domain
CAP	Cysteine-rich secretory proteins, antigen 5, and pathogenesis-related 1 proteins
SDS-PAGE	Sodium dodecyl sulfate-polyacrylamide gel electrophoresis
Sol i 1	Venom Allergen 1, phospholipase A1, from the fire ant *Solenopsis invicta*
Sol i 2	Venom Allergen 2 from the fire ant *S. invicta*
Sol i 3	Venom Allergen 3, SCP/CRISP-like protein, from the fire ant *S. invicta*
Sol i 4	Venom Allergen 4 from the fire ant *S. invicta*
