# Supplementary material for: Bottom-Up Proteomic Analysis of Polypeptide Venom Components of the Giant Ant Dinoponera Quadriceps
Source: Toxins (Basel). 2019 Jul 29;11(8):448. doi: 10.3390/toxins11080448 (PMC6722740; doi:10.3390/toxins11080448)
Supplement: Supplementary file 1 [file toxins-11-00448-s001.pdf]

Supplementary Materials: Bottom-up Proteomic Analysis of Polypeptide Venom Components of the Giant Ant *Dinoponera quadriceps*

Douglas Oscar Ceolin Mariano, Úrsula Castro de Oliveira, André Junqueira Zaharenko, Daniel Carvalho Pimenta, Gandhi Rádis-Baptista and Álvaro Rossan de Brandão Prieto-da-Silva

Contig164_B2-2 *D. quadriceps* venom dipeptidyl peptidase 4 [Corrected] 5'3' Frame +1 insertion G position 359

```
   1 gcgatatattagtctctgtcggacgtactcgcgggatcggcgaggcgtcgcgctgcagcggtatttcgaacatc
   1                     5' UTR                    Start M  A  G  H  T  V  L  P  L  L
  76 gtccccggagctcgcgatccacgaatcggcccgacggtgatccacATGGCAGGACACACGGTTCTCCCACTTTTG
  11 A  L  F  S  C  L  F  V  I  G  Q  T  R  T  V  S  R  V  D  E  S  S  I  D  V
 151 GCGCTGTTCAGCTGTCTGTTCGTCATCGGGCAAACACGAACAGTCTCGCGCGTCGACGAAAGCAGCATCGATGTT
  36 P  V  Y  K  D  S  D  V  K  K  P  F  L  L  E  E  I  Y  F  H  F  Y  T  P  F
 226 CCCGTTTACAAGGATTCCGATGTAAAAAAGCCGTTCCTCTTGGAGGAGATTTATTTCCACTTCTACACACCGTTC
  61 G  F  N  G  T  W  I  S  D  D  E  I  L  M  E  N  T  I  L  G  D  I  T  K  Y
 301 GGCTTCAACGGCACGTGGATCTCCGACGATGAAATCTTAATGGAGAACACGATTCTCGGCGATATCACCAAGTAC
  86 N  V  M  T  G  E  T  T  T  M  F  D  G  K  N  L  P  L  E  Y  K  F  G  S  G
 376 AACGTAATGACCGGGGAGACGACAACGATGTTCGACGGGAAGAACTTACCGCTGGAATACAAATTCGGTTCGGGA
 111 T  F  S  P  D  K  Q  N  I  L  F  T  Y  N  V  K  H  V  F  R  H  S  K  I  M
 451 ACTTTCTCACCCGACAAACAGAACATTCTCTTTACTTACAACGTCAAACACGTGTTCAGGCACTCGAAGATCATG
 136 S  I  V  V  Y  D  V  K  R  G  T  F  E  K  V  A  N  E  S  R  V  L  L  A  K
 526 AGTATCGTGGTGTATGACGTGAAACGGGGCACGTTCGAAAAGGTCGCGAACGAATCCCGTGTCTTGCTGGCGAAG
 161 W  L  P  N  G  N  G  L  V  Y  V  R  N  N  D  I  Y  H  A  V  F  K  D  G  K
 601 TGGTTGCCGAACGGCAACGGATTGGTCTACGTTCGCAATAACGACATTTATCACGCGGTTTTCAAGGACGGCAAG
 186 S  I  V  R  R  L  T  K  S  G  K  S  G  I  V  F  N  G  I  S  D  W  V  Y  E
 676 AGCATAGTGCGAAGACTGACGAAGAGCGGCAAATCCGGAATTGTGTTCAACGGAATTTCAGACTGGGTGTATGAA
 211 E  E  V  Y  A  S  S  T  A  M  W  H  S  P  H  G  R  Y  L  A  F  A  T  F  N
 751 GAGGAAGTATACGCTTCGTCGACCGCGATGTGGCATTCGCCGCATGGACGGTATCTGGCCTTCGCAACCTTCAAC
 236 D  T  Q  V  R  D  M  E  Y  S  H  Y  G  V  P  G  S  L  R  D  Q  Y  P  T  Q
 826 GACACTCAAGTGAGGGATATGGAGTATTCCCATTACGGCGTGCCCGGTTCTCTTAGGGACCAATACCCCACGCAA
 261 V  K  L  K  Y  P  K  V  G  T  P  N  P  I  V  S  L  S  V  I  D  L  T  D  P
 901 GTGAAGCTCAAGTATCCCAAGGTGGGCACGCCGAATCCGATAGTGTCCTTGTCGGTGATCGATCTGACCGATCCA
 286 S  S  E  A  V  T  L  E  A  P  V  H  V  V  G  S  D  N  I  L  Y  T  V  S  W
 976 TCGTCGGAGGCGGTCACGTTAGAAGCGCCCGTCCACGTGGTCGGCAGCGACAACATCCTGTATACCGTGAGCTGG
 311 W  N  L  T  H  V  T  A  T  W  T  N  R  V  Q  N  Q  S  Q  L  V  M  Y  D  A
1051 TGGAACCTGACGCACGTGACCGCAACGTGGACGAACCGCGTGCAGAATCAATCCCAGCTGGTGATGTACGATGCG
 336 Q  G  T  A  K  L  V  W  Y  D  E  E  R  E  G  W  L  Q  P  N  S  P  V  K  V
1126 CAGGGCACCGCTAAGCTTGTTTGGTACGACGAGGAGAGGGAAGGCTGGCTTCAGCCGAACAGCCCTGTGAAAGTC
 361 G  N  Y  A  L  L  L  R  Q  E  D  S  G  T  S  A  G  K  F  R  H  I  V  R  Y
1201 GGCAATTACGCGTTGCTCCTACGCCAAGAGGATTCCGGCACGAGCGCCGGGAAGTTCCGGCACATAGTGAGATAC
 386 E  C  D  D  G  R  F  T  S  R  V  D  L  T  P  G  P  S  E  V  H  S  I  Q  A
1276 GAGTGCGACGACGGCCGGTTCACTTCGCGAGTGGATCTCACGCCCGGTCCCTCCGAGGTGCACTCGATCCAGGCC
 411 V  D  S  R  R  G  R  V  Y  Y  F  A  T  A  P  G  E  P  S  R  R  N  L  Y  S
1351 GTCGATTCGCGCAGAGGCCGAGTTTACTACTTCGCTACGGCACCGGGCGAGCCAAGCCGGAGGAATTTGTATTCG
 436 V  A  L  D  A  S  Q  N  P  T  C  I  S  C  E  Q  H  T  P  E  G  N  K  C  A
1426 GTGGCTCTGGACGCGAGCCAGAACCCGACTTGCATATCCTGCGAGCAGCATACGCCGGAAGGGAACAAATGTGCC
 461 Y  A  Y  A  S  F  S  S  R  M  S  H  Y  A  L  S  C  S  G  P  D  L  L  A  V
1501 TACGCGTACGCGTCTTTTTCAAGCCGGATGTCGCATTACGCTCTCAGCTGCTCCGGTCCCGATTTACTCGCGGTG
 486 T  I  H  D  E  N  H  R  R  I  T  T  W  E  D  N  K  L  I  R  D  M  L  S  K
1576 ACCATACACGACGAGAACCATCGGCGAATCACGACGTGGGAAGATAACAAATTGATAAGGGACATGCTGTCGAAG
 511 K  L  M  P  L  Q  R  D  F  N  V  T  V  N  G  Y  D  S  R  V  R  L  F  L  P
1651 AAATTGATGCCGTTGCAGAGAGATTTCAACGTGACCGTGAACGGCTATGATTCCAGAGTCAGGTTATTCCTGCCG
 536 P  D  F  D  E  T  E  S  Y  P  M  L  V  Y  V  Y  G  G  P  N  S  A  R  I  V
1726 CCGGACTTCGACGAGACGGAATCGTACCCCATGCTGGTATACGTTTACGGCGGCCCGAACAGTGCCAGGATCGTC
 561 D  V  A  K  F  G  Y  E  H  Y  M  T  T  N  K  R  V  I  Y  A  W  I  D  G  R
1801 GACGTAGCTAAGTTCGGATACGAGCATTACATGACGACCAACAAACGCGTCATTTACGCGTGGATCGACGGACGA
 586 G  S  A  F  K  G  S  K  M  L  F  E  I  Y  R  R  I  G  T  V  E  V  E  D  T
1876 GGATCAGCCTTCAAGGGCAGTAAGATGCTGTTCGAGATATACAGGCGCATAGGAACCGTCGAGGTCGAAGACACG
 611 I  A  V  T  K  I  L  Q  E  R  Y  S  W  I  D  P  D  R  T  G  I  W  G  W  S
1951 ATCGCTGTTACCAAGATTCTACAGGAACGGTACAGCTGGATCGACCCGGACAGGACCGGTATATGGGGCTGGAGT
 636 Y  G  G  F  T  T  G  M  V  L  A  T  D  V  E  S  V  F  K  C  G  I  S  V  A
2026 TACGGTGGGTTCACCACCGGCATGGTTCTCGCCACGGACGTAGAATCCGTGTTCAAGTGCGGCATTTCAGTCGCG
 661 P  V  T  S  W  I  Y  Y  D  S  I  Y  T  E  R  F  M  G  L  P  T  V  D  D  N
2101 CCCGTAACTTCTTGGATCTATTACGACTCCATTTACACAGAGCGGTTCATGGGGTTGCCAACCGTGGACGACAAC
 686 L  A  G  Y  N  A  T  D  I  S  R  R  V  R  G  I  R  G  K  K  Y  M  L  I  H
2176 CTAGCCGGCTACAACGCCACGGATATCTCGCGACGAGTGCGAGGGATACGCGGCAAGAAGTACATGCTGATACAC
 711 G  T  E  D  D  N  V  H  Y  Q  Q  A  M  A  L  A  K  S  L  E  R  R  D  I  L
2251 GGTACCGAGGACGACAACGTGCATTATCAGCAGGCGATGGCGCTCGCCAAGTCGCTGGAGCGACGGGACATCCTA
 736 F  E  Q  V  T  Y  T  D  E  V  H  A  L  L  R  V  S  P  H  L  Y  H  T  M  D
2326 TTTGAACAGGTCACGTACACGGACGAGGTGCACGCTCTCTTGCGCGTGTCCCCCCACCTCTATCACACGATGGAC
 761 K  F  W  S  E  C  F  G  L  D  R  T  R  *Stop              3' UTR
2401 AAATTTTGGAGCGAGTGCTTCGGCCTGGATCGCACCCGCTGAcctggtgctccgccgcgacgagtcgacgcggat
2476 gagaatcgttcgcagcgagccgaggattcgctcgtccccgaacgcaccagtttctccgcgaaacgatcgagaata
2551 cactctcggggatccgttcggacgatcctccgtgacattacgtcgactctcttctttgtggagaatatttgacgt
2626 aacgtgtcctcggcaacgtcaatgacgacgggatgttccccgtggaaattcaggacgacgacagttccgagatac
2701 actctgtatccacgcagatatcctgcagacacgtccgaacgcattaaaactcgatcgtacaaagaaaaaaaaaaa
2776 gaaaaaaaa
```

◆ Active Site (Charge relay system)

polyadenylation signal

Figure S1. Structural nucleotide organization of venom dipeptidyl peptidase-4 of *D. quadriceps* expressed transcript contig164_B2-2. The product of this transcript is found in SDS-PAGE, gel band 1. (Figure 2, in the main text). In purple, part of the 5'UTR is shown, followed by the start codon that initiates the signal peptide of 21 amino acids predicted by SignalP 5.0 (in yellow). Followed by the coding region of a mature protein with 752 amino acids (pink), the active site is indicated by the catalytic triad (green diamonds). Followed by the stop codon, the 3 'UTR region (purple) and the polyadenylation signal (ATTAAA), indicated by a yellow box.

Contig75_B2-2 *D. quadriceps* Glucose dehydrogenase [FAD, quinone] 3'5' Frame 2

```
                                        5' UTR
   1 tcgggaaatatttgacctgatcgacagtacggcgcgtcgtatcgatcaggtcaaatatttcccgagaaaaagctc
  76 tttaagtcgtggaaaaaagcaatgtcgtttgattaaacataaggatttgccggcggtgccgattggattagtaaa
 151 tttcacactgacgagagaaaagagaaaagacgttataagttgaaagaaaaatacttgatatttgtacctgtaatc
 226 attataaaatattatttggttacgataaatacgtaaaatgaaacaaataatttccttgtgagaaaattatctttc
 301 gcttcaaacaaattttcatttaaaataaaggtgcaaggataagagggcatcgataagaggaagctcgaggaagg
   1 Start M  G  I  E  S  V  L  A  G  G  L  T  T  A  S  T  G  F  S  W  F  Y  P  V
 376 taaaATGGGCATCGAGAGCGTTCTGGCAGGGGGTTTGACCACGGCCTCGACCGGGTTCTCTTGGTTCTATCCAGT
  25  L  A  A  A  L  V  Y  F  H  Y  E  V  M  D  N  E  A  P  P  I  N  V  P  S  E
 451 CCTCGCCGCGGCGCTCGTCTACTTCCATTACGAAGTTATGGACAACGAGGCGCCGCCAATTAACGTGCCGTCTGA
  50  V  L  L  H  S  Y  D  F  I  V  I  G  G  G  S  A  G  S  V  V  A  N  R  L  S
 526 GGTGCTATTACACTCGTATGATTTCATAGTGATAGGCGGCGGCTCGGCAGGTTCCGTGGTGGCGAATCGTCTTTC
  75  E  I  E  K  W  N  V  L  L  L  E  A  G  G  D  E  T  E  I  S  D  V  P  L  L
 601 TGAGATCGAGAAGTGGAATGTGCTGCTGTTGGAAGCGGGTGGCGACGAGACGGAGATCTCGGATGTGCCCTTGCT
 100  A  G  Y  L  Q  L  S  Q  L  D  W  Q  Y  K  T  E  P  Q  I  G  A  C  L  A  M
 676 CGCCGGCTATCTCCAGCTCAGTCAATTGGACTGGCAATACAAGACCGAGCCGCAGATCGGTGCCTGTCTAGCGAT
 125  E  N  G  Q  C  N  W  P  R  G  K  V  I  G  G  S  S  V  L  N  Y  M  L  Y  L
 751 GGAGAACGGTCAGTGCAACTGGCCGAGGGGCAAGGTGATCGGAGGCTCGTCGGTCTTAAATTACATGCTTTACTT
 150  R  G  N  R  R  D  Y  N  T  W  E  E  Q  G  N  P  G  W  G  W  R  E  V  L  H
 826 GCGCGGCAATCGGCGGGATTACAACACATGGGAGGAGCAGGGCAATCCTGGTTGGGGCTGGCGCGAGGTCTTGCA
 175  Y  F  K  K  S  E  D  N  K  N  P  Y  L  V  R  T  P  Y  H  A  D  G  G  Y  L
 901 CTATTTCAAGAAGTCCGAGGACAACAAAAATCCATACCTGGTCCGCACGCCGTACCACGCCGATGGCGGATACCT
 200  T  V  Q  E  A  P  W  H  T  P  L  A  A  A  F  V  Q  A  G  Q  E  M  G  Y  E
 976 GACGGTGCAGGAGGCGCCTTGGCACACGCCTCTGGCAGCGGCCTTCGTCCAAGCCGGTCAAGAAATGGGCTATGA
 225  N  R  D  I  N  G  E  Y  Q  T  G  F  M  I  V  Q  G  T  L  R  R  G  S  R  C
1051 GAATCGCGATATCAACGGCGAGTATCAAACCGGCTTTATGATCGTGCAGGGCACACTCAGGCGCGGCAGCCGTTG
 250  S  A  A  K  A  F  L  R  P  V  R  L  R  K  N  L  H  V  A  M  H  A  H  V  T
1126 CTCCGCCGCGAAGGCCTTCCTCAGGCCGGTGCGGCTCAGGAAGAACCTGCACGTGGCGATGCACGCGCACGTGAC
 275  K  V  L  I  H  P  K  S  K  R  T  Y  G  V  E  F  V  R  D  G  K  V  F  R  I
1201 CAAAGTGCTGATACATCCAAAGTCGAAGCGCACTTACGGCGTGGAGTTCGTCAGAGACGGCAAGGTGTTCCGTAT
 300  R  A  N  K  E  V  I  V  S  G  G  A  I  N  S  P  Q  L  L  M  L  S  G  I  G
1276 ACGCGCGAACAAGGAGGTGATCGTATCCGGTGGCGCGATCAACTCGCCCCAACTGCTGATGTTGTCCGGAATAGG
 325  P  K  E  H  L  Q  E  F  G  I  P  V  I  Q  N  S  K  V  G  Q  N  L  Q  D  H
1351 GCCAAAGGAGCATCTGCAGGAATTCGGGATCCCCGTGATACAGAATTCGAAGGTTGGGCAAAATCTTCAGGACCA
 350  V  G  L  G  G  L  T  F  M  V  N  Q  E  I  S  M  V  Q  K  R  L  E  N  T  Q
1426 CGTCGGCCTAGGTGGACTCACATTCATGGTGAATCAGGAGATCTCCATGGTGCAGAAGCGGCTGGAGAACACCCA
 375  V  V  I  Q  Y  A  V  L  G  D  G  P  L  T  V  P  G  G  V  E  G  V  A  F  V
1501 GGTAGTGATACAATACGCCGTGCTGGGCGACGGGCCACTGACGGTGCCGGGCGGCGTCGAGGGCGTTGCTTTCGT
 400  N  S  K  Y  A  N  A  S  L  D  Y  P  D  I  E  L  H  F  V  S  G  S  I  N  S
1576 CAACTCCAAGTACGCGAACGCATCGCTGGACTATCCCGACATCGAGTTGCACTTCGTCTCCGGCTCGATCAACTC
 425  D  G  G  T  Q  L  R  K  V  H  G  L  T  K  Q  F  Y  D  N  V  Y  G  P  I  N
1651 GGACGGTGGCACTCAGCTACGTAAAGTGCACGGGCTCACGAAGCAATTCTATGACAACGTTTACGGGCCGATCAA
 450  N  K  D  T  W  S  V  I  P  M  L  L  R  P  K  S  R  G  V  I  K  L  R  S  K
1726 CAACAAGGACACGTGGAGTGTGATCCCCATGCTGCTGCGGCCCAAAAGCCGCGGTGTCATCAAGCTGCGCAGCAA
 475  N  P  F  D  H  P  L  I  Y  P  N  Y  F  K  E  P  E  D  M  A  T  L  V  E  G
1801 GAACCCGTTCGACCACCCTCTCATCTATCCGAACTACTTCAAGGAACCGGAGGACATGGCCACGCTGGTGGAAGG
 500  V  K  I  S  V  A  L  S  R  T  A  A  F  R  R  F  G  S  E  L  Y  S  M  Q  F
1876 AGTGAAGATCAGCGTGGCCTTGAGCAGGACAGCCGCCTTCCGACGATTCGGCAGCGAGCTGTACTCGATGCAGTT
 525  P  G  C  K  H  I  P  M  Y  T  D  P  Y  W  E  C  M  I  R  H  Y  S  A  T  I
1951 CCCAGGGTGCAAGCATATCCCTATGTATACGGATCCTTACTGGGAATGCATGATTCGACATTACAGTGCGACGAT
 550  Y  H  P  V  G  T  C  K  M  G  E  Y  W  D  P  D  A  V  V  D  P  Q  L  R  V
2026 ATACCACCCGGTTGGCACATGCAAGATGGGAGAGTACTGGGATCCCGACGCTGTGGTCGACCCGCAGCTACGGGT
 575  Y  G  V  A  G  L  R  V  I  D  A  S  I  M  P  N  L  V  S  G  N  T  N  A  P
2101 TTACGGCGTGGCTGGGCTGCGCGTGATCGACGCCTCGATCATGCCGAATCTCGTAAGTGGAAACACGAATGCGCC
 600  T  I  M  I  G  E  K  G  A  D  M  I  K  E  Y  W  L  K  R  K  H  P  P  E  F
2176 CACGATCATGATCGGCGAGAAAGGCGCCGACATGATCAAGGAGTATTGGCTGAAACGGAAGCACCCGCCTGAGTT
 625  S  K  Q  *Stop                          3' UTR
2251 TAGTAAACAGTAAttaggcgggcacgatcaactacggacagacgtgagcgcgctacaacgagagtgataagaatg
2326 ttaccaatgcgcggtgaaggaggttttgcaatttccgggatatttcgagcgaatttcttccggacaacccgaagt
2401 gtcgagaacgcatgattcgttgtgtacatatatgtgtgtctaagttgtcatgcttataatgcgacgcaataaaat
2476 attagtattatattccgcaatatagagatataggtgtaaaatatgtaagaaaaataataatctctccgcggacag
2551 acgaagttaccaatctctacacgatttgttctaatcgcgagatcagtcgcctgcaattattctcggtcgatttca
2626 cgtcgaatatcgtcgagacacatgattctaattccctggaattatccgcgtgaattacacgtcaataggcgattt
2701 attaaacgttaaatataatattaaatgataaaaaaaacagaacgatctaacatccgcgcggaaagtaaatttctt
2776 ttcatcgtttgataaaaataataagtcggtcaattaaacaagtacgcaagcattttcaaggatggaaataaaaa
2851 ctgctgacgtttggataataaaaaaaaaaa                            polyadenylation signal
```

Active Site (Proton acceptor)

Nucleotide binding, FAD

Figure S2. Glucose dehydrogenase precursor [FAD, quinone] encoded by Contig75_B2-2. The mature peptide was found in the venom proteomics, according to explanation in the main text. The cDNA encodes a precursor with 627 amino acids, the mature part of which is indicated in salmon. The nucleotide binding site is indicated by the pink box. The 5 'and 3'-untranslated regions (UTRs) are indicated in purple. The polyadenylation signal is indicated by the yellow box. The proton acceptor of the active site indicated by a green diamond.

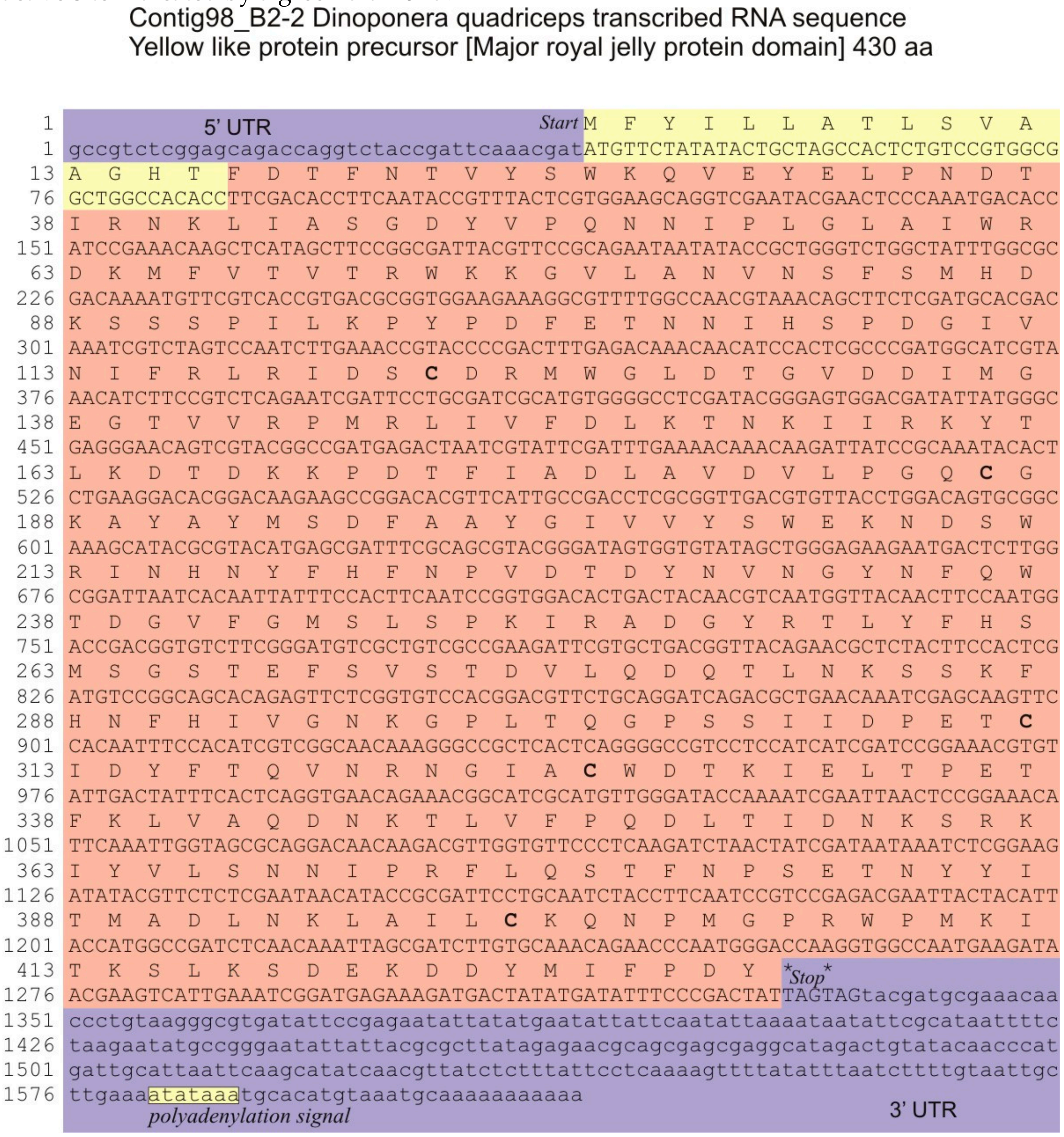

Figure S3. Structural nucleotide organization of the precursor of *D. quadriceps* MRJP-like venom protein. The contig98_B2-2 cDNA code for major royal jelly protein-like in giant ant venom gland.The mature toxins were found in the venom by the proteomic analysis (this study). Non-coding regions 5 'and 3'-UTRs are indicated in purple. The predicted signal peptide is shown in yellow. The coding region for the mature protein (detailed in the main text) is indicated in beige/orange. Start and stop codons are indicated with asterisks. The putative polyadenylation site is indicated by the yellow box.

Contig27_B2-2 transcribed RNA sequence, phospholipase A1 2-like, Dinoponera quadriceps

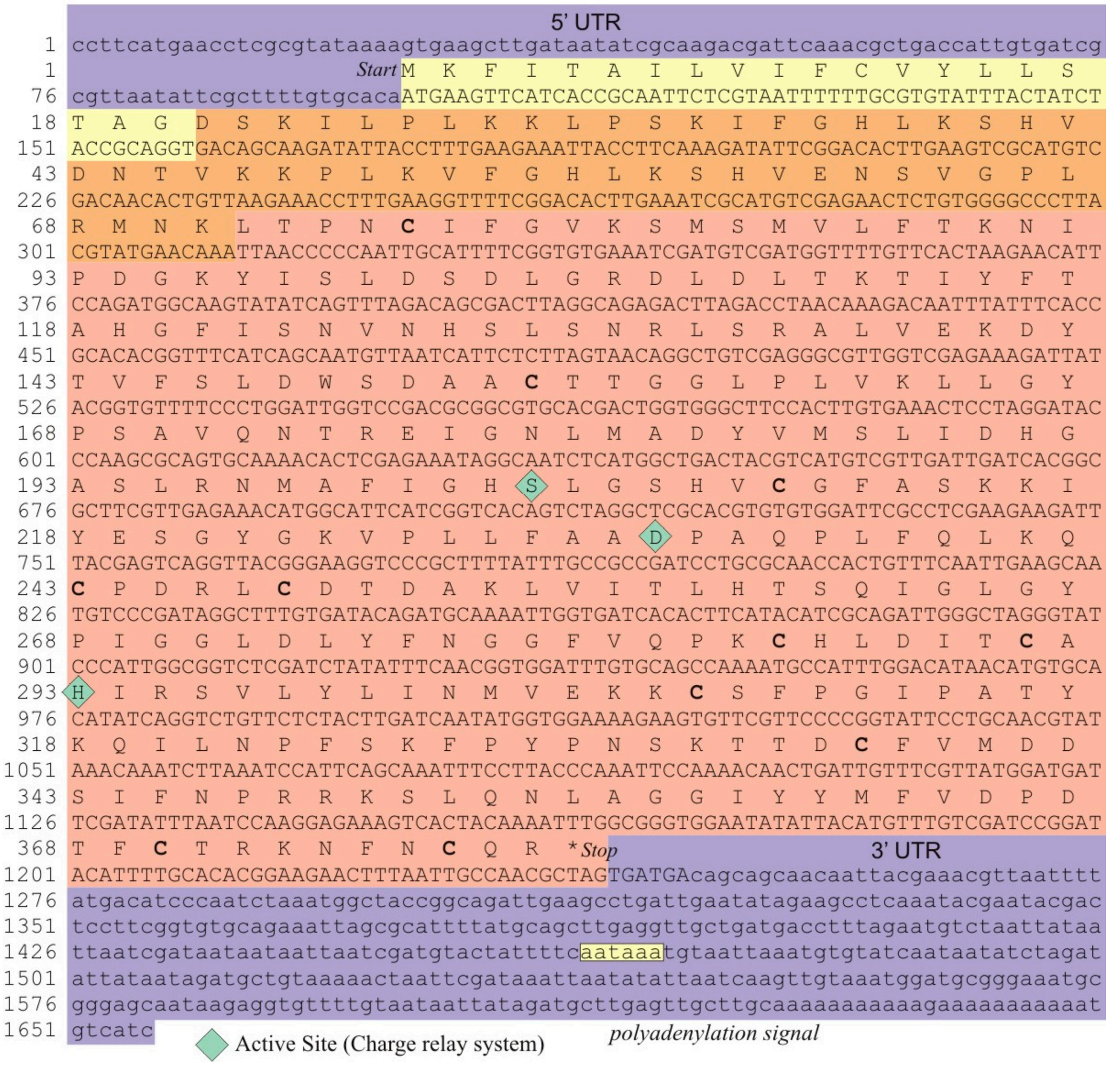

Figure S4. Structural organization of the Contig27_B2-2 cDNA that code for phospholipase A1 in the *D. quadriceps* venom. The full-length precursor is 379-amino acid residues long. A signal peptide with 20 amino acids, as predicted by SignalP 5.0, is indicated in yellow. The 5 'and 3'-untranslated regions are indicated in purple. The mature sequence is indicated in light pink and the putative propeptide is visible in the region indicated in orange. The mature peptide has 11 cysteines of which 10 are conserved among the PLA1s from the venom of hymenopterans. The mature peptide is found in the venom as detected by proteomic analysis (this study). The amino acids in the active site are indicated by green diamonds. The predicted polyadenylation signal is indicated by a yellow box

Contig385_B2-2 *Dinoponera quadriceps* venom Hyaluronidase

```
                                 5'UTR
   1 agcgcactaacgacgttaaccgtctctggtcagcgggggaggtgcgcgaatcttcgccgagagacgagcattg
  76 tttaacatcaccttaccaattgactgtttagtggcgccttgacaccgcggtcgactcccgggacgtctgatcgcc
 151 agtttctagttcaccagcgggaaactcctcaccaccgaatctccgtagtagagccgccacgaaacttcatcgtcg
   1             M  L  P  V  R  N  S  L  I  T  A  F  A  T  V  L  V  A  G  F  F
 226 agttcgagcacgATGCTACCAGTTCGAAACTCTCTGATTACTGCCTTCGCGACCGTCCTGGTCGCCGGTTTCTTC
  22 G  S  D  A  T  T  L  K  E  G  D  P  T  P  F  D  V  Y  W  N  V  P  T  F  M
 301 GGCTCAGATGCGACGACCCTCAAGGAAGGCGATCCGACCCCGTTTGACGTTTACTGGAACGTGCCCACTTTCATG
  47 C  H  K  Y  G  M  K  F  E  E  L  K  D  F  G  I  R  Q  N  A  M  D  E  F  R
 376 TGTCACAAGTATGGCATGAAATTCGAGGAGCTGAAGGATTTCGGCATTCGTCAGAACGCCATGGACGAGTTCCGT
  72 G  E  K  I  A  I  L  Y  D  P  G  M  F  P  A  L  L  T  D  K  N  G  H  E  T
 451 GGCGAAAAGATCGCGATTCTTTACGACCCGGGGATGTTCCCGGCATTGTTGACCGATAAGAACGGACATGAGACG
  97 S  R  N  G  G  V  P  Q  E  G  D  L  K  K  H  L  Q  V  F  E  E  H  L  V  S
 526 AGCAGGAACGGCGGTGTGCCGCAAGAAGGCGATCTGAAAAAACACCTCCAGGTGTTCGAGGAGCATTTAGTCTCG
 122 Q  I  P  N  E  S  F  S  G  V  G  V  I  D  F  E  S  W  R  P  I  F  R  Q  N
 601 CAGATCCCCAACGAGTCGTTTAGCGGCGTCGGCGTGATAGATTTCGAGAGCTGGCGGCCCATCTTCCGGCAAAAT
 147 W  A  S  L  E  P  Y  K  T  R  S  F  E  V  E  R  K  R  H  P  F  W  S  D  A
 676 TGGGCCTCTCTCGAGCCCTATAAGACCCGGTCGTTCGAGGTGGAACGTAAGAGGCATCCCTTCTGGAGCGACGCG
 172 A  I  K  K  E  A  K  R  R  F  E  K  Y  G  R  M  F  M  E  E  T  L  K  T  A
 751 GCCATCAAGAAGGAAGCAAAGCGTCGTTTCGAGAAGTACGGTAGGATGTTCATGGAGGAGACTCTGAAGACGGCG
 197 K  K  L  R  S  K  A  A  W  G  Y  Y  G  Y  P  Y  C  F  N  Q  T  P  G  Q  R
 826 AAGAAGCTGCGGTCTAAGGCCGCGTGGGGTTACTACGGCTATCCTTATTGCTTCAATCAGACACCCGGCCAGCGC
 222 S  A  H  C  D  R  K  T  V  V  E  N  D  E  M  S  W  L  F  T  L  E  D  L  H
 901 AGCGCGCATTGCGATCGCAAAACCGTGGTGGAGAATGACGAAATGTCTTGGCTGTTCACGCTCGAGGACCTGCAC
 247 A  P  S  V  Y  L  R  L  E  I  K  E  E  D  R  P  G  F  V  K  G  R  V  S  E
 976 GCGCCTTCCGTGTACTTGAGACTGGAGATCAAGGAGGAAGATCGTCCGGGCTTCGTTAAGGGCAGAGTATCGGAG
 272 A  L  R  L  Q  S  K  S  P  N  A  Q  R  T  L  P  Y  Y  W  F  K  Y  Q  D  R
1051 GCGCTGCGGCTACAGTCGAAGAGCCCGAACGCACAACGGACCTTGCCTTATTACTGGTTTAAGTACCAAGATAGA
 297 R  D  E  F  L  S  K  K  D  T  Y  N  T  I  D  T  I  A  G  L  G  A  N  G  L
1126 AGAGATGAATTCCTGAGCAAGAAAGACACATATAACACGATTGACACGATAGCCGGCCTCGGCGCCAACGGTTTG
 322 I  I  W  G  S  S  D  D  V  N  A  E  Q  K  C  K  N  L  Q  N  H  V  K  D  V
1201 ATCATTTGGGGCAGCTCCGATGACGTGAACGCCGAGCAGAAGTGCAAGAACCTGCAGAACCACGTGAAAGATGTC
 347 L  G  P  T  I  K  R  F  T  T  T  Q  *               3'UTR
1276 CTGGGGCCGACGATAAAGCGATTCACAACCACGCAGTAAtgcgttctaatcgggagagccgaggatcgcgcgttg
1351 acctgtctcaagtacaaatgacgttaccgacttacattccgcttctaccatcgtggattaaatctaatcggcggt
1426 cgactcaacgaaattgaccaatcgcattggtcagttcagctaatcagctgatgcgattcgttaattccgttgagt
1501 ttaccgtcagtaagatttaaactatgatgatagaaactggcgttatccattagttcgcgaattaacccccagtt
1576 cccaatacttccccgtaacacttgcaccttagatttcgaagcaatcgtgtactcgaacgggatatgacgtattta
1651 taaatatcgattagaaaacatacgatctcgatatatatatcgtcgaagagagttatataaagaagacctgaggaa
1726 gacacgggtgagtcattgggaaccgaggggtaaaagagagactgaatagtaacgatcgcacgagagaaagagaga
1801 gagagagagaagagagaagagaa
```

Figure S5. The venom hyaluronidase precursor, transcript contig385_B2-2, from *D. quadriceps* venom gland. Venom hyaluronidase was identified in proteomic analysis (this study) and confirmed the presence of the transcript product in the venom. Structurally, the untranslated regions, 5'-UTR and 3'-UTR, are indicated in purple. The coding region comprises a precursor of 368 amino acids, containing a signal peptide of 25 amino acids, as indicated in yellow, and a mature protein with 343 amino acids, as initiated in rose. This venom component belongs to the family of glycosyl hydrolases 56. The conserved cysteine residues, that form intramolecular disulfide bonds are indicated in bold and the proton donor of the active site is indicated by a green diamond. These characteristics are typical of a catalytically active hyaluronidase, in *D. quadriceps* venom.

Contig12_B2-2 *Dinoponera quadriceps* Major Allergen-3 Precursor (CAP/SPC)

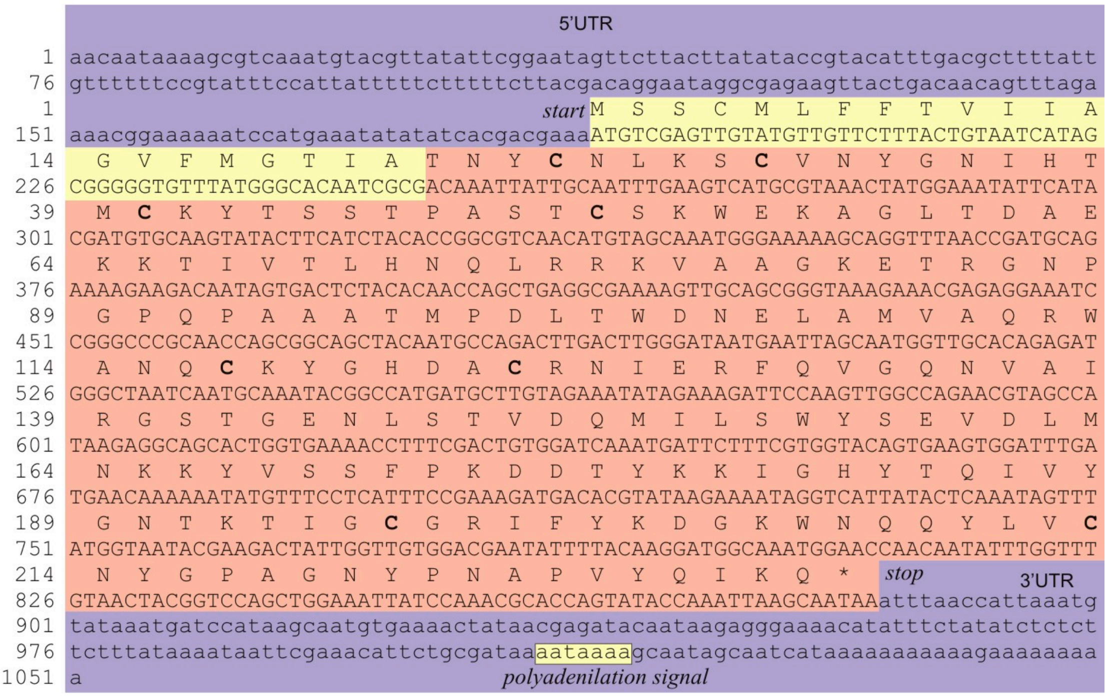

Figure S6. Structural nucleotide organization of transcript contig12_B2-2 that code for major allergen-3 in *D. quadriceps*. The major allergen-3 was identified in the proteomic, in this study, as one of the predominant components in the crude venom. In orange, the mature polypeptide is evidenced with its conserved 8 cysteine residues (in bold). This protein structure is typical of the V5-SCP/CAP (CRISP) family. Highlighted in purple, is indicate the 5′- and 3′-untranslated regions. In yellow the putative signal peptide as predicted by SignalP5.0. In yellow, the polyadenylation signal is boxed.

Contig8_B2-2 Ant Venom Allergen 2/4 (OBP/PBP)precursor *Dinoponera quadriceps*

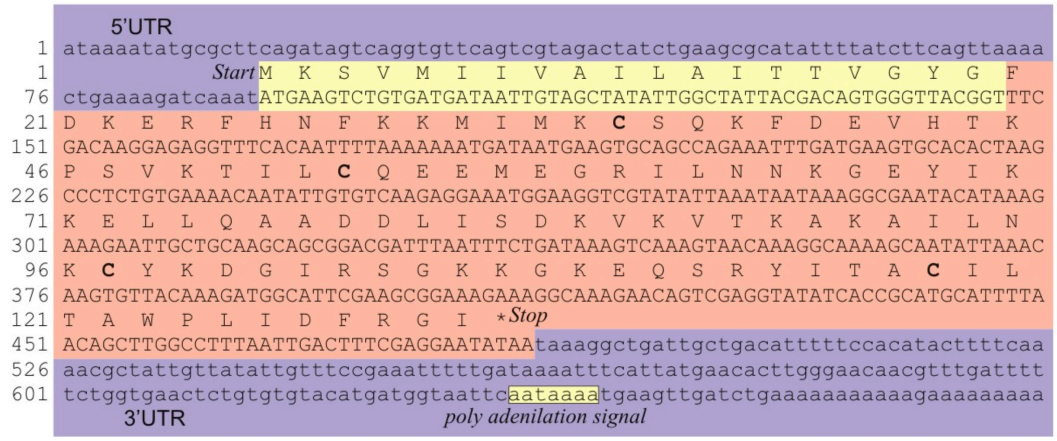

Figure S7. Structural organization of *D. quadriceps* venom gland transcript for Venom allergen 2/4 (Contig29_B2-2) and respective protein precursor product. The sequence is 675 bp-long. In purple the untranslated regions (5 'and 3' UTRs) are shown. In yellow the signal peptide region predicted by SpecialP 5.0 (19aa) and in pink the mature peptide (112aa). A polyadenylation signal is indicated by a yellow box. Four conserved cysteines in the Ant venom Allergen 2/4 family are indicated in bold type and may form two disulfide bonds by similarity.

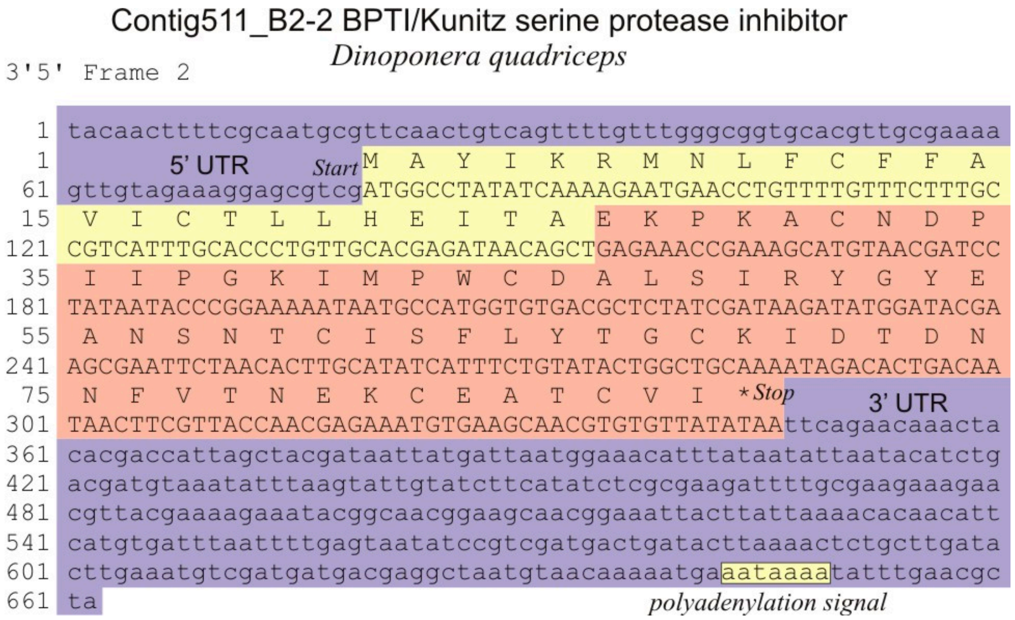

Figure S8. The *D. quadriceps* venom component BPTI/Kunitz-serine protease inhibitor. The structural organization of transcript Contig511_B2-2 from the *D. quadriceps* venom gland, identified by peptide mass fingerprint mass spectrometry analysis is shown. The signal peptide is shown in yellow; The mature part in salmon. The yellow box indicates the polyadenylation signal. In purple the untranslated regions.

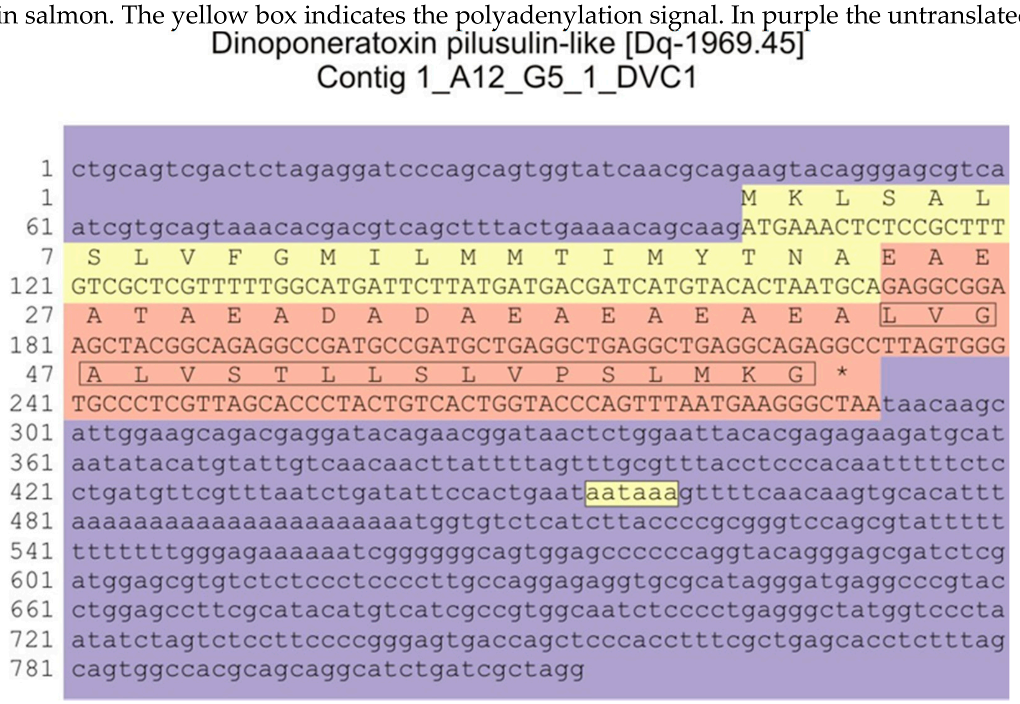

(**A**)

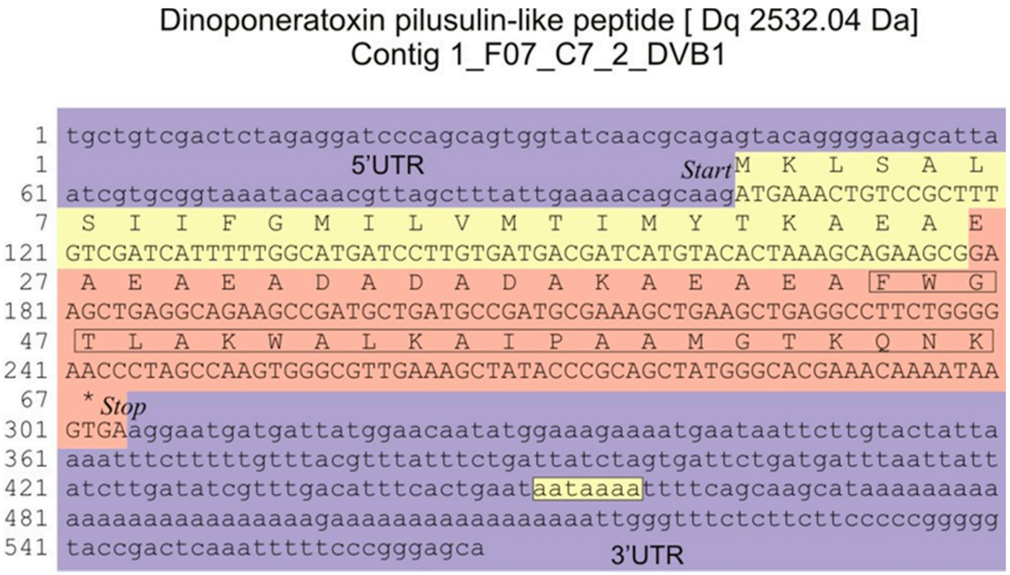
Dinoponeratoxin pilusulin-like peptide [Dq 2532.04 Da]
Contig 1_F07_C7_2_DVB1
1 tgctgtcgactctagaggatcccagcagtggtatcaacgcagagtacaggggaagcatta
1 5'UTR Start M K L S A L
61 atcgtgcggtaaatacaacgttagctttattgaaaacagcaagATGAAACTGTCCGCTTT
7 S I I F G M I L V M T I M Y T K A E A E
121 GTCGATCATTTTTGGCATGATCCTTGTGATGACGATCATGTACACTAAAGCAGAAGCGGA
27 A E A E A D A D A D A K A E A E A F W G
181 AGCTGAGGCAGAAGCCGATGCTGATGCCGATGCGAAAGCTGAAGCTGAGGCCTTCTGGGG
47 T L A K W A L K A I P A A M G T K Q N K
241 AACCCTAGCCAAGTGGGCGTTGAAAGCTATACCCGCAGCTATGGGCACGAAACAAAATAA
67 * Stop
301 GTGAaggaatgatgattatggaacaatatggaaagaaaatgaataattcttgtactatta
361 aaatttctttttgtttacgtttatttctgattatctagtgattctgatgatttaattatt
421 atcttgatatcgtttgacatttcactgaataataaaattttcagcaagcataaaaaaaaa
481 aaaaaaaaaaaaaaaagaaaaaaaaaaaaaaaaattgggtttctcttcttcccccggggg
541 taccgactcaaatttttcccgggagca
3'UTR

(**B**)

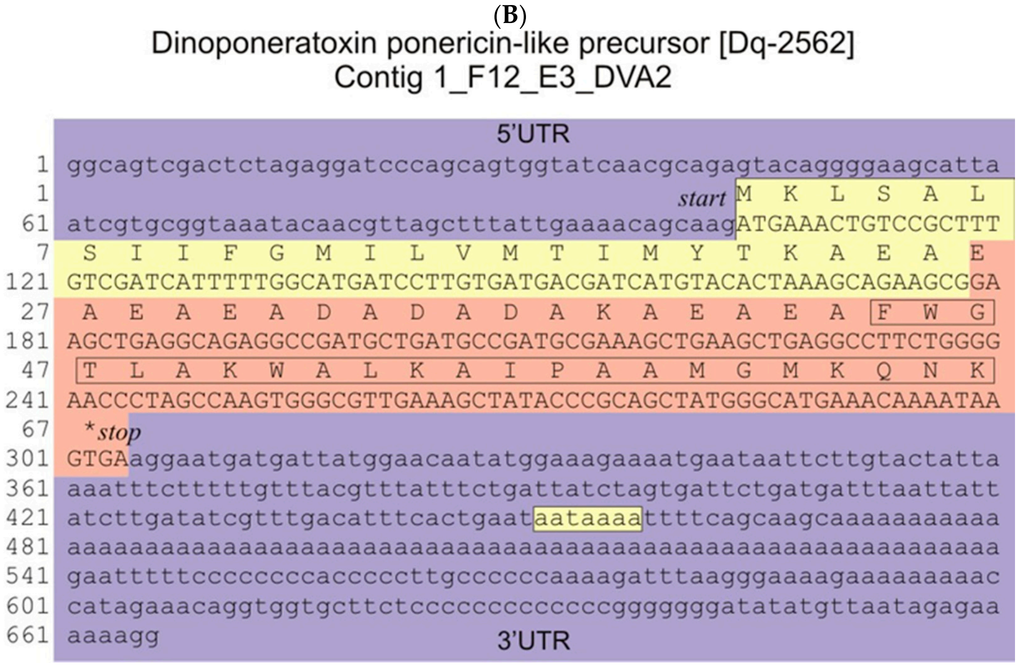
Dinoponeratoxin ponericin-like precursor [Dq-2562]
Contig 1_F12_E3_DVA2
5'UTR
1 ggcagtcgactctagaggatcccagcagtggtatcaacgcagagtacaggggaagcatta
1 start M K L S A L
61 atcgtgcggtaaatacaacgttagctttattgaaaacagcaagATGAAACTGTCCGCTTT
7 S I I F G M I L V M T I M Y T K A E A E
121 GTCGATCATTTTTGGCATGATCCTTGTGATGACGATCATGTACACTAAAGCAGAAGCGGA
27 A E A E A D A D A D A K A E A E A F W G
181 AGCTGAGGCAGAGGCCGATGCTGATGCCGATGCGAAAGCTGAAGCTGAGGCCTTCTGGGG
47 T L A K W A L K A I P A A M G M K Q N K
241 AACCCTAGCCAAGTGGGCGTTGAAAGCTATACCCGCAGCTATGGGCATGAAACAAAATAA
67 * stop
301 GTGAaggaatgatgattatggaacaatatggaaagaaaatgaataattcttgtactatta
361 aaatttctttttgtttacgtttatttctgattatctagtgattctgatgatttaattatt
421 atcttgatatcgtttgacatttcactgaataataaaattttcagcaagcaaaaaaaaaaa
481 aaaaaaaaaaaaaaaaaaaaaaaaaaaaaaaaaaaaaaaaaaaaaaaaaaaaaaaaaaaa
541 gaattttccccccccaccccccttgccccccaaaagatttaagggaaaagaaaaaaaaac
601 catagaaacaggtggtgcttctcccccccccccccgggggggatatatgttaatagagaa
661 aaaagg
3'UTR

(**C**)

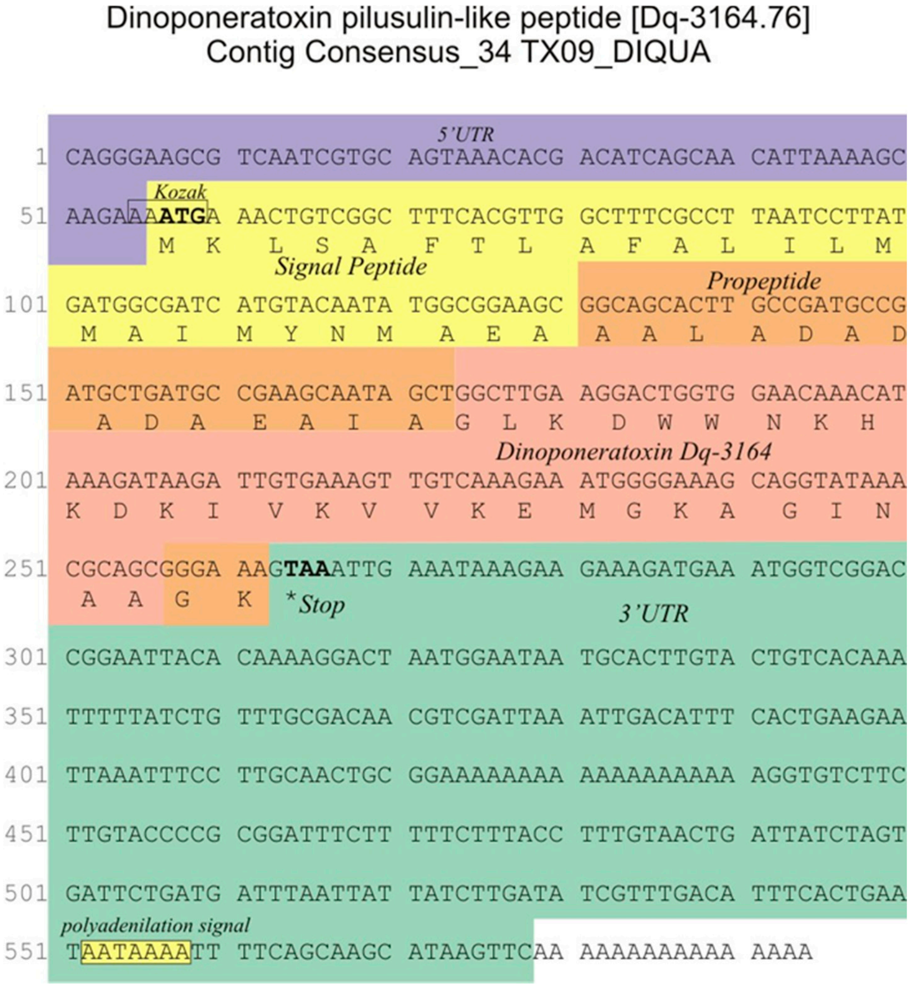

(**D**)

Figure S9. *D. quadriceps* venom gland transcript precursors for dinoponeratoxins (pilosulin- and ponericin-like peptides) with their respective peptide products, identified by proteomic analysis. The structural organization of transcrips and respective mature peptide, are as follow: **A.** Contig 1_A12_G5_1_DVC1 Pilosulin-like Precursor Peptide [Dq-1969]; **B**. Contig 1_F07_C7_2_DVB1 Pilosulin-like Precursor Peptide [Dq- 2532]; **C.** Contig Consensus 34 Pilosulin-like Precursor Peptide; **D.** Contig 1_F12_E3_DVA2 Pilosulin-like precursor peptide [Dq-2562]. In purple, the transcript untranslated regions (UTR) are shown; in yellow the signal of peptides as predicted by the SignalP 5.0 algorithm and in salmon the regions of the pro-peptide. A yellow box indicates the signal of polyadenylation convenience in every 3´-UTR sequence. The amino acid sequence of the mature peptide, of which fragments were identified by mass spectrometry analysis (this study) are indicated by open box or underlined.

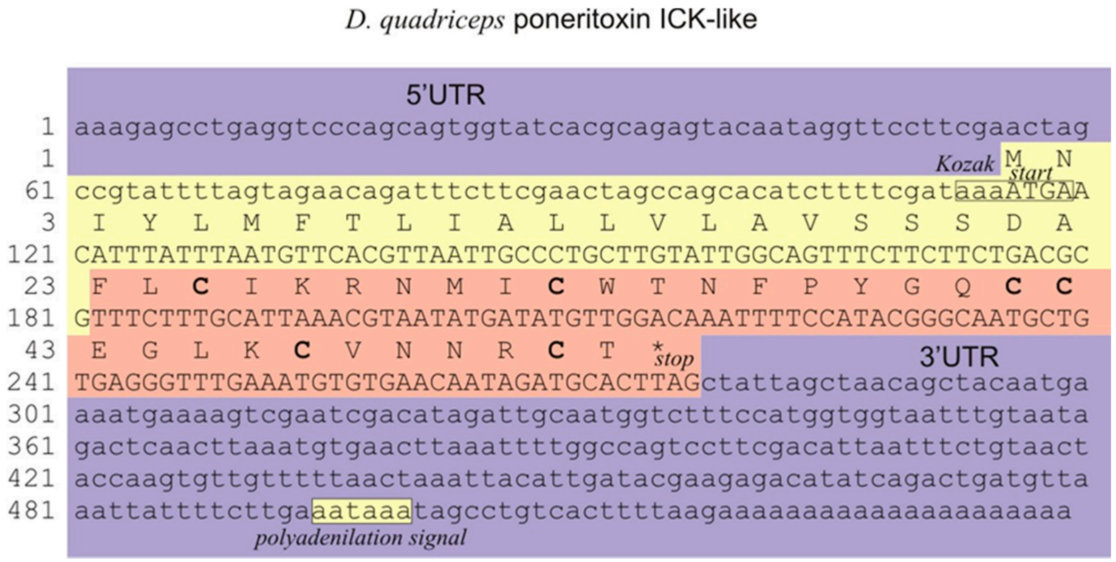

(**A**)

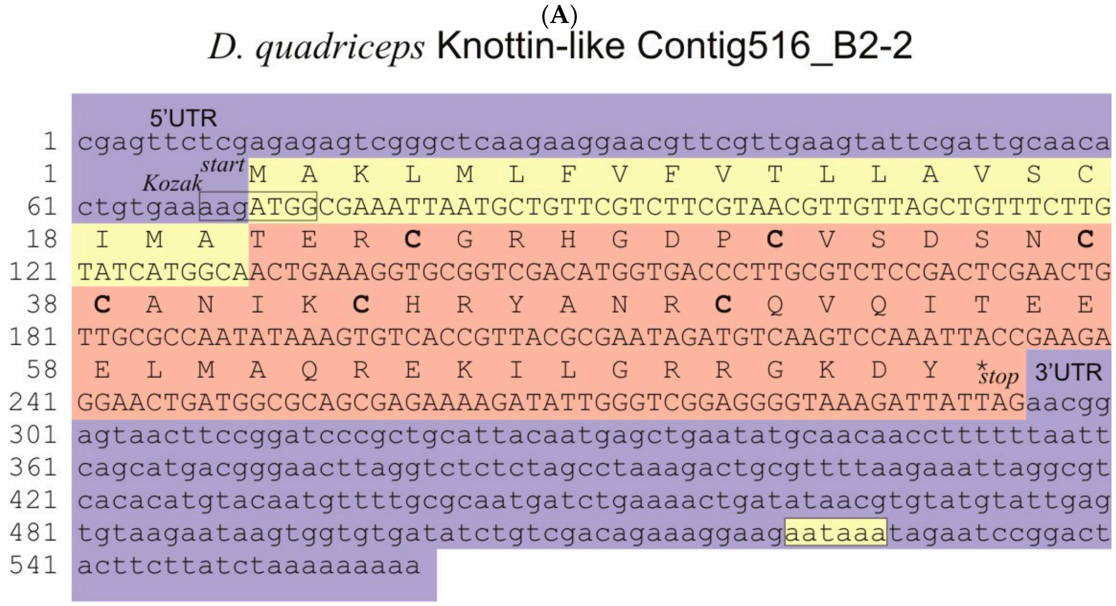

(**B**)

Figure S10. Structural organization of transcripts encoding knottin (ICK)-like toxins from the venom gland of D. quadriceps, of which identifiers of mature peptides were found in the venom proteomics. A. Contig 1_D07_A11_4_DVB1 coding for an ICK-like toxin (U1-poneritoxin-Dq5a) similar to neurotoxins from honey bee and Conus sea snail. B. structural organization of the transcript contig516_B2-2 gi|578895399| encoding a toxin similar to neurotoxins from honey bee and the ponerine ant O. monticola. The 5 'and 3'-UTR transcript termini are shown in purple. The signal peptides were predicted by SignalP 5.0 and are indicated by yellow boxes. The mature peptides, of which the full sequences matched several peptide fragments identified by in-solution proteomics, are enlightened in pink color. The polyadenylation signal (AATAA) are indicated by yellow boxes. The start codon and the stop codon are indicated the conserved cysteines which may form disulfide bonds are indicated in bold.
